# Influence of nonlocal on a rotating thermoelastic medium with diffusion and double porosity

**DOI:** 10.1038/s41598-025-97334-3

**Published:** 2025-05-07

**Authors:** Doaa M. Salah, A. M. Abd-Alla, S. M. M. El-Kabeir

**Affiliations:** 1 Department of Mathematics, Faculty of Science, Sohag University, Sohag, Egypt; 2 Department of Mathematics, Faculty of Science, Aswan University, Aswan, Egypt

**Keywords:** Nonlocal thermoelastic, Diffusion, Double porosity, Rotation, Normal mode, Materials science, Mathematics and computing

## Abstract

In this paper, we have studied the thermodynamical interactions in a two-dimensional thermoelastic diffusion in homogeneous isotropic medium with nonlocal, rotation and double porosity in the context of Green–Lindsay theory. A thermal load was applied to the medium’s outer free surface. By employing the Lame’s potential and normal mode technique, we derived analytical expressions for field quantities such as thermal stresses, displacement components, temperature field, equilibrated stresses and diffusion. Numerical computations conducted using MATLAB programming for a specific material, illustrated the results. Several graphs are presented to examine the effects of time, nonlocal parameter, double porosity and rotation. Furthermore, comparisons are made between CT, LS, and GL theories. The theoretical and numerical results were found to be in close agreement. Computer-simulated numerical results are presented through graphical representations illustrating the physical quantities for both the nonlocal thermoelastic half-space with double porosity under the influence of rotation. Additionally, the results offer valuable insights for fields such as geophysics and biomedical engineering, where the interaction between multiple fields is critical. Comparative plots further illustrate the nonlocal parameter, time, rotation and porosity effects on the wave propagation properties.

**Supplementary Information:**

The online version contains supplementary material available at 10.1038/s41598-025-97334-3.

## Introduction

The study of double porosity elastic materials has gained significant attention due to their unique structural characteristics and diverse applications. These materials exhibit two distinct levels of porosity: macro porosity, characterized by large visible pores, and micro porosity, formed by tiny fissures or cracks. This dual-pore structure leads to the presence of two independent pressure fields one associated with the liquid in the macro pores and another governing the fluid behavior in the micro pores. A fissured rock, for instance, serves as a prime example of a double porosity medium, consisting of porous blocks separated by a network of fractures or fissures. Despite extensive research in the field of porous materials, existing studies often overlook the complex interactions between the macro and micro structures, particularly in the context of elasticity and fluid dynamics. Traditional models fail to comprehensively capture the independent yet interdependent pressure fields within such materials. Furthermore, thermoelasticity theories, both coupled and uncoupled, have struggled to align with physical experiments, highlighting fundamental gaps in theoretical modeling. This study aims to bridge these gaps by developing a refined theoretical framework that accurately represents the mechanics of double porosity elastic materials. By critically analyzing existing literature, we identify deficiencies in current models and propose an improved approach that integrates both micro and macro porosity effects more effectively. Our research introduces a novel methodology that enhances the predictive capabilities of double porosity models, offering new insights into the behavior of fissured materials under various conditions. The implications of this research extend beyond theoretical advancements. Double porosity materials play a crucial role in several engineering and geophysical applications, including petroleum extraction, hydrogeology, and biomedical engineering. A more precise understanding of their behavior can lead to improved reservoir modeling, enhanced material design, and optimized fluid transport in biological systems. By addressing the existing limitations in double porosity modeling, this study contributes to both scientific progress and practical innovations in various fields. In this framework, Biot^1^ motivated the heat conduction equation and introduced the dynamic theory of thermoelasticity (CD theory) based on Fourier’s law of heat. The CD theory explored the rapid propagation of thermal waves. As research advanced, Lord and Shulman^2^ introduced a key idea by incorporating a relaxation time into the heat equation. Concurrently, Green and Lindsay^3^ extended the heat conduction equation by introducing two relaxation times. Their work led to the development of the generalized thermoelasticity theory, which has since been widely explored by various authors^4–7^. Although numerous studies have investigated disturbances in a thermoelastic medium with diffusion, there has been no attempt to examine two-dimensional disturbances in a rotating double porous thermoelastic medium with diffusion under the G–L theory as a result of applied nonlocal and thermal loads. Therefore, the uniqueness of this study lies in its novel approach to investigating how field quantities such as normal stress, normal displacement, equilibrated stresses, temperature, and concentration are influenced by the various parameters in the medium due to the application of thermal load. The theory of generalized thermoelasticity with rotation and diffusion finds applications in various fields, including earthquake engineering, astronautics, aeronautics, and soil dynamics. Heat and mass diffusion processes are crucial in numerous engineering applications, such as satellite issues, re-entry of space vehicles, and aircraft landings on water or land. There is significant interest in diffusion processes for manufacturing integrated circuits, integrated resistors, semiconductor substrates, and MOS transistors. Studying diffusion also helps improve oil extraction conditions. In many fields, particularly astronomy and related areas, rotation is a commonly observed phenomenon. The effects of rotation on velocity are critical for designing acoustic sensors. In particular, velocity shifts due to rotation have been used in gyroscopes. A set of seismometers, sensitive to rotational inputs, was developed at a geophysical institute in Prague. Rotation effects are also considered in many sensitive and accurate devices used in modern medical applications, such as “RP” (rotation by pressure sensor) and “RC” (capacitive sensing rotation) sensors. Additionally, since rotational movements significantly influence album prediction devices, it was found that these effects should be considered, as accurate measurement devices must measure real transitional acceleration and distortions caused by rotational movements. The theory of non-local elasticity, emphasizing that the applied force at a given point in a continuous body depends not only the point on the strain but also on strains due to stress in surrounding regions was studied by Eringen’s^8,9^. Mondal et al.^10^ analyzed the model of dual-phase-lag (DPL) thermo-elastic solid material with voids employing Eringen’s non-local elasticity model. The introduction of a double porous structure to the thermoelastic medium has garnered the attention of many engineers, seismologists, and scientists due to its applications in geophysics, material science, bone mechanics, pharmaceuticals, and the medical device industry. Studying dynamical interactions in a thermoelastic solid under the effects of diffusion and rotation, with additional parameters like double porosity, is crucial for various technological and geophysical applications^11–17^. Thermodiffusion in an elastic solid arises from the coupling of strain, temperature, and mass diffusion fields, as well as heat and mass exchange with the environment. Using a coupled thermoelastic model, Nowacki^18–20^ proposed the theory of thermoelastic diffusion, which predicts infinite velocities for the propagation of thermoelastic signals. The study of wave propagation and thermoelastic interactions in complex materials has been a focal point of research due to its wide-ranging applications in engineering, geophysics, and material sciences. Various factors, including rotation, nonlocal effects, fractional order behavior, and electromagnetic influences, play crucial roles in determining the mechanical and thermal responses of these materials. Recent studies have explored the propagation and reflection of thermoelastic waves in rotating nonlocal fractional order porous media under the influence of Hall currents^21^. Additionally, comparative analyses have been conducted to investigate temperature-dependent characteristics and non-local behavior in submerged microstretch thermoelastic media using multiple models^22^. Further research has examined the effects of internal heat sources, rotation, magnetic fields, and initial stress on p-wave propagation within photothermal semiconducting media^23^. The impact of magnetic fields and initial stress on rotating photothermal semiconductor media has also been explored, particularly in conjunction with ramp-type heating and internal heat sources^24^. These investigations contribute to the development of a fully coupled system within the generalized thermoelastic theory for semiconductor materials^25^. By critically analyzing these studies, this research aims to provide a comprehensive understanding of thermoelastic wave behavior in various advanced materials. The findings have significant implications for designing materials with tailored mechanical and thermal properties, optimizing wave propagation in semiconductor devices, and enhancing applications in geophysics and aerospace engineering.

In the present work, we have studied the thermodynamical interactions in a two-dimensional rotating thermoelastic medium with nonlocal, diffusion and double porosity in the context of Green–Lindsay theory. A thermal load is applied at the outer free surface of the medium. The normal mode technique is employed to derive the analytic expressions of the field quantities such as stresses, displacement components, temperature field and diffusion. Despite existing research on thermoelastic media with diffusion, this study uniquely explores thermomechanical disturbances in a rotating thermoelastic half-space with diffusion and double porosity under the impact of nonlocal. Numerical computations are performed with the help of MATLAB software for a specific material for illustrating the results. Results indicate significant influences of these parameters, and the model includes comparisons between models (CT–LS–GL) as special cases. Comparisons of the physical quantities are shown in Figures to study the effects of time, double porosity, rotation, thermal load and diffusion. The implications of these findings extend to diverse engineering sub-domains, including soil dynamics, seismology, geophysics, and earthquake engineering. The obtained results depict that the influences of these parameters are all significant.

## Formulation of the problem

**Fig. 1:**
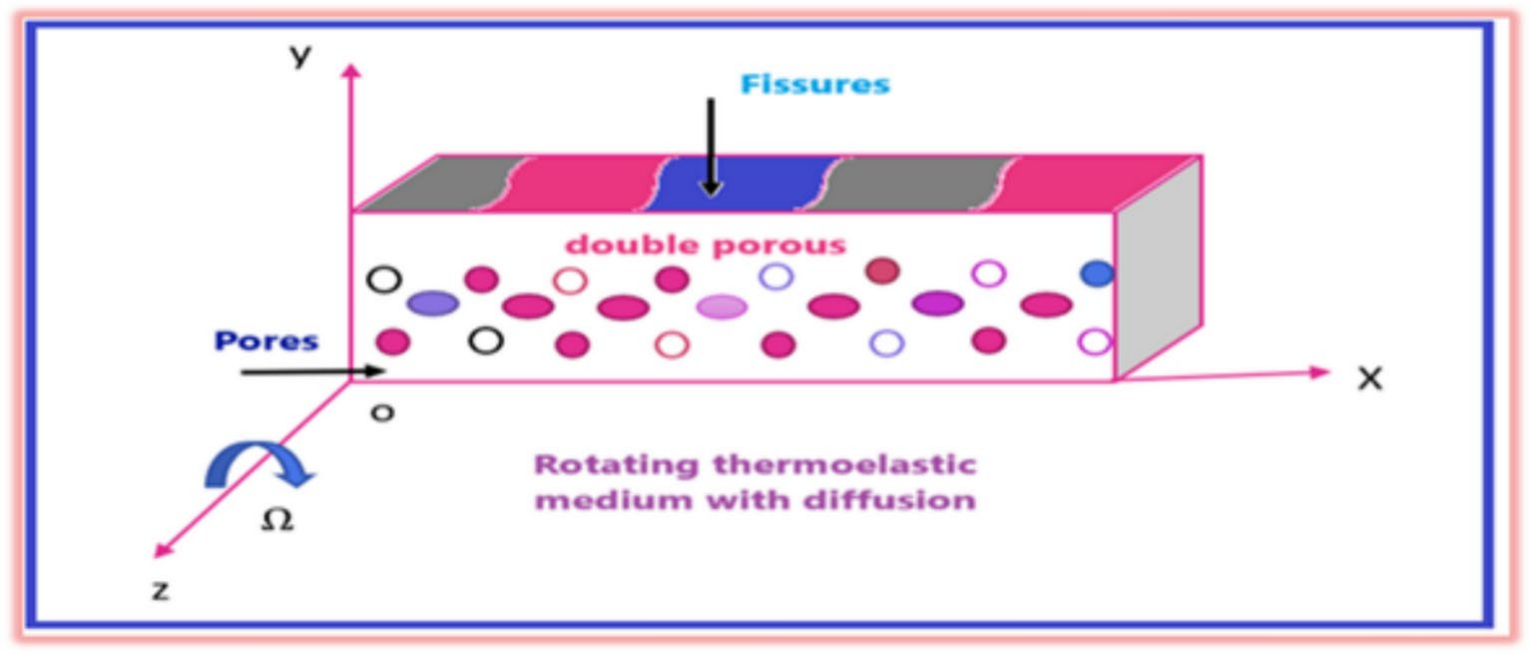
Geometry of the problem.

We examine a rotating double porous homogeneous thermoelastic solid medium using the GL model under the effect of a nonlocal parameter and diffusion. The coordinate system has its origin at point O on the horizontal surface, with the axis directed downward into the solid half-space as shown in Fig. 1. The fundamental field quantities depend solely on *x*, *y*, and *t*, and they remain unaffected by the variable. A disturbance in the *x–y* plane occurs at the surface, gradually diminishing as *x* approaches infinity. The displacement equation of motion in the rotating plane has two extra terms: centripetal acceleration $\vec{\Omega } \wedge \left( {\vec{\Omega } \wedge {\vec{\text{u}}}} \right)$ caused by just time-varying motion, and Coriolis acceleration $\left( {2{\vec{\Omega }} \wedge \overrightarrow {{{\dot{\text{u}}}}} } \right)$ caused by a moving frame reference such that the displacement components are *(u = u (x, y, t)) and (v = v (x, y, t))*.

The equations of motion in the absence of the body forces are

1 $$\begin{gathered} \rho \left( {1 - L_{N}^{2} \nabla^{2} } \right)\frac{{\partial^{2} u}}{{\partial t^{2} }} - \rho {\Omega }^{2} u + 2\rho {\Omega }\frac{\partial v}{{\partial t}} = \mu \left( {\frac{{\partial^{2} u}}{{\partial x^{2} }} + \frac{{\partial^{2} u}}{{\partial y^{2} }}} \right) + \left( {\lambda + \mu } \right)\left( {\frac{{\partial^{2} u}}{{\partial x^{2} }} + \frac{{\partial^{2} v}}{\partial x\partial y}} \right) \hfill \\ - \beta_{1} \left( {1 + \tau_{1} \frac{\partial }{\partial t}} \right)\frac{\partial \theta }{{\partial x}} - \beta_{2} \left( {1 + \tau^{1} \frac{\partial }{\partial t}} \right)\frac{\partial C}{{\partial x}} + b\frac{{\partial q_{1} }}{\partial x} + d\frac{{\partial q_{2} }}{\partial x}, \hfill \\ \end{gathered}$$

2 $$\begin{gathered} \rho \left( {1 - L_{N}^{2} \nabla^{2} } \right)\frac{{\partial^{2} u}}{{\partial t^{2} }} - \rho {\Omega }^{2} u + 2\rho {\Omega }\frac{\partial v}{{\partial t}} = \mu \left( {\frac{{\partial^{2} v}}{{\partial y^{2} }} + \frac{{\partial^{2} v}}{{\partial y^{2} }}} \right) + \left( {\lambda + \mu } \right)\left( {\frac{{\partial^{2} v}}{{\partial y^{2} }} + \frac{{\partial^{2} u}}{\partial x\partial y}} \right) \hfill \\ - \beta_{1} \left( {1 + \tau_{1} \frac{\partial }{\partial t}} \right)\frac{\partial \theta }{{\partial y}} - \beta_{2} \left( {1 + \tau^{1} \frac{\partial }{\partial t}} \right)\frac{\partial C}{{\partial y}} + b\frac{{\partial q_{1} }}{\partial y} + d\frac{{\partial q_{2} }}{\partial y}, \hfill \\ \end{gathered}$$

The heat conduction equation is

3 $$\begin{gathered} K\left( {\frac{{\partial^{2} \theta }}{{\partial x^{2} }} + \frac{{\partial^{2} \theta }}{{\partial y^{2} }}} \right) = \rho c_{e} \frac{\partial }{\partial t}\left( {1 + \tau_{0} \frac{\partial }{\partial t}} \right)\theta + \beta_{1} T_{o} \left( {\frac{{\partial^{2} u}}{\partial x\partial t} + \frac{{\partial^{2} v}}{\partial y\partial t}} \right) + a_{c} T_{o} \frac{\partial }{\partial t}\left( {1 + \tau^{o} \frac{\partial }{\partial t}} \right)C \hfill \\ + \gamma_{1} T_{o} \left( {1 + \tau_{o} \frac{\partial }{\partial t}} \right)\frac{{\partial q_{1} }}{\partial t} + \gamma_{2} T_{o} \left( {1 + \tau_{o} \frac{\partial }{\partial t}} \right)\frac{{\partial q_{2} }}{\partial t}, \hfill \\ \end{gathered}$$

The equilibrated stress equations of motions are

4 $$\begin{gathered} - b\left( {\frac{\partial u}{{\partial x}} + \frac{\partial v}{{\partial y}}} \right) + \alpha \left( {\frac{{\partial^{2} q_{1} }}{{\partial x^{2} }} + \frac{{\partial^{2} q_{1} }}{{\partial y^{2} }}} \right) - \alpha_{1} q_{1} + b_{1} \left( {\frac{{\partial^{2} q_{2} }}{{\partial x^{2} }} + \frac{{\partial^{2} q_{2} }}{{\partial y^{2} }}} \right) - \alpha_{3} q_{2} + \gamma_{1} \theta + \upsilon C \hfill \\ = k_{1} (1 - L_{N} \nabla^{2} )\frac{{\partial^{2} q_{1} }}{{\partial t^{2} }}, \hfill \\ \end{gathered}$$

5 $$\begin{gathered} - d\left( {\frac{\partial u}{{\partial x}} + \frac{\partial v}{{\partial y}}} \right) + b_{1} \left( {\frac{{\partial^{2} q_{1} }}{{\partial x^{2} }} + \frac{{\partial^{2} q_{1} }}{{\partial y^{2} }}} \right) - \alpha_{3} q_{1} + \gamma \left( {\frac{{\partial^{2} q_{2} }}{{\partial x^{2} }} + \frac{{\partial^{2} q_{2} }}{{\partial y^{2} }}} \right) - \alpha_{2} q_{2} + \gamma_{2} \theta + mC \hfill \\ = k_{2} \left( {1 - L_{N}^{2} \nabla^{2} } \right)\frac{{\partial^{2} q_{2} }}{{\partial t^{2} }} \hfill \\ \end{gathered}$$

The stresses components are

6 $$\sigma_{xx} = 2\mu \frac{\partial u}{{\partial x}} + \lambda \left( {\frac{\partial u}{{\partial x}} + \frac{\partial v}{{\partial y}}} \right) - \beta_{1} \left( {1 + \tau_{1} \frac{\partial }{\partial t}} \right)\theta - \beta_{2} \left( {1 + \tau^{1} \frac{\partial }{\partial t}} \right)C + bq_{1} + dq_{2} ,$$

7 $$\sigma_{yy} = 2\mu \frac{\partial v}{{\partial y}} + \lambda \left( {\frac{\partial u}{{\partial x}} + \frac{\partial v}{{\partial y}}} \right) - \beta_{1} \left( {1 + \tau_{1} \frac{\partial }{\partial t}} \right)\theta - \beta_{2} \left( {1 + \tau^{1} \frac{\partial }{\partial t}} \right)C + bq_{1} + dq_{2} ,$$

8 $$\sigma_{xy} = \mu \left( {\frac{\partial u}{{\partial y}} + \frac{\partial v}{{\partial x}}} \right),$$

The equilibrated stress corresponding to pores

9 $$\sigma_{x} = \alpha \frac{{\partial q_{1} }}{\partial x} + b_{1} \frac{{\partial q_{2} }}{\partial x},$$

10 $$\sigma_{y} = \alpha \frac{{\partial q_{1} }}{\partial y} + b_{1} \frac{{\partial q_{2} }}{\partial y},$$

The equilibrated stress corresponding to fissures

11 $$\chi_{x} = b_{1} \frac{{\partial q_{1} }}{\partial x} + \gamma \frac{{\partial q_{2} }}{\partial x},$$

12 $$\chi_{y} = b_{1} \frac{{\partial q_{1} }}{\partial y} + \gamma \frac{{\partial q_{2} }}{\partial y},$$

The equation of diffusion

13 $$\begin{aligned} D_{c} b_{c} \left( {1 + \tau^{1} \frac{\partial }{\partial t}} \right)\left( {\frac{{\partial^{2} C}}{{\partial x^{2} }} + \frac{{\partial^{2} C}}{{\partial y^{2} }}} \right) & = D_{c} \beta_{2} \left( {\frac{{\partial^{2} }}{{\partial x^{2} }} + \frac{{\partial^{2} }}{{\partial y^{2} }}} \right)\left( {\frac{\partial u}{{\partial x}} + \frac{\partial v}{{\partial y}}} \right) \\ & \quad + D_{c} a_{c} \left( {1 + \tau_{1} \frac{\partial }{\partial t}} \right)\left( {\frac{{\partial^{2} \theta }}{{\partial x^{2} }} + \frac{{\partial^{2} \theta }}{{\partial y^{2} }}} \right) + D_{c} \upsilon \left( {\frac{{\partial^{2} q_{1} }}{{\partial x^{2} }} + \frac{{\partial^{2} q_{1} }}{{\partial y^{2} }}} \right) \\ & \quad + D_{c} m\left( {\frac{{\partial^{2} q_{2} }}{{\partial x^{2} }} + \frac{{\partial^{2} q_{2} }}{{\partial y^{2} }}} \right) + \frac{\partial C}{{\partial t}}, \\ \end{aligned}$$

Here b, d, $\alpha$, $\alpha_{1}$, $\alpha_{2}$, $\alpha_{3}$, $v$, $m$, $b_{1}$, $b_{2}$, γ, $\gamma_{1}$, $\gamma_{2}$ are constitutive coefficient, $a_{c}$ is measure of thermodiffusion effect, $D_{c}$ is the thermoelastic diffusion constant, $k_{1}$ and $k_{2}$ are the equilibrated inertia coefficients, $b_{c}$ is measure of diffusive effect, $\sigma_{i}$ and $\chi_{i}$ are the equilibrated stresses corresponding to pores and fissures respectively, and $\beta_{1} = \left( {3\lambda + 2\mu } \right)\alpha_{t}$, $\beta_{2} = \left( {3\lambda + 2\mu } \right)\alpha_{c}$. The dimensionless variables listed below can be used to improve the transformation of the main quantities in the dimensionless case:

14 $$\begin{gathered} \left( {\acute{x} ,\acute{y} ,\acute{u} ,\acute{v} } \right) = \frac{{\omega _{1}}}{{c_{1}}}~\left( {x,y,u,v} \right),\left( {\acute{q_{1}} ,\acute{q_{2}} } \right) = \frac{{k_{1} \omega _{1} ^{2} }}{{\alpha _{1} }}~\left( {q_{1} ,q_{2} } \right),\sigma _{{ij}}^{\prime } = \frac{1}{{\beta _{2} T_{o} ~}}\sigma _{{ij}} ,~\theta ^{\prime } = \frac{\theta }{{T_{o} }},~c_{1}^{2} = \frac{{\lambda + 2\mu }}{\rho }, \hfill \\ C^{\prime} = \frac{C}{{T_{o}}},~\acute{\Omega} = \frac{\Omega }{{\omega _{1}}},\left( {~\sigma _{i}^{\prime} ,\chi _{i} ^{\prime}} \right) = \frac{{c_{1}}}{{\alpha \omega _{1} ~}}\left( {\sigma _{{ij}} ,\chi _{i}} \right),(t^{\prime} ,~\tau _{0}^{\prime} ,~\tau _{1}^{\prime} ,\tau ^{{1\prime}} ,\tau ^{{o\prime}} ) = \omega _{1} (t,~\tau _{0} ,\tau _{1} ,\tau ^{1} ,\tau ^{o} ), \hfill \\ \omega _{1} = \frac{{\rho c^{*} c_{1}^{2}}}{K}. \hfill \\ \end{gathered}$$

To simplify the presentation of the governing Eqs. (1)–(13) with Eq. (14) dashes are removed. Consequently, it generates

15 $$\begin{aligned} \rho \left( {1 - a_{0} L_{N}^{2} \nabla^{2} } \right)\frac{{\partial^{2} u}}{{\partial t^{2} }} - \rho {\Omega }^{2} u + 2\rho {\Omega }\frac{\partial v}{{\partial t}} & = a_{1} \left( {\frac{{\partial^{2} u}}{{\partial x^{2} }} + \frac{{\partial^{2} u}}{{\partial y^{2} }}} \right) + a_{2} \left( {\frac{{\partial^{2} u}}{{\partial x^{2} }} + \frac{{\partial^{2} v}}{\partial x\partial y}} \right) \\ & \quad - a_{3} \left( {1 + \tau_{1} \omega_{1} \frac{\partial }{\partial t}} \right)\frac{\partial \theta }{{\partial x}} - a_{4} \left( {1 + \tau^{1} \omega_{1} \frac{\partial }{\partial t}} \right)\frac{\partial C}{{\partial x}} \\ & \quad + a_{5} \frac{{\partial q_{1} }}{\partial x} + a_{6} \frac{{\partial q_{2} }}{\partial x}, \\ \end{aligned}$$

16 $$\begin{aligned} \rho \left( {1 - a_{0} L_{N}^{2} \nabla^{2} } \right)\frac{{\partial^{2} v}}{{\partial t^{2} }} - \rho {\Omega }^{2} v + 2\rho {\Omega }\frac{\partial u}{{\partial t}} & = a_{1} \left( {\frac{{\partial^{2} v}}{{\partial x^{2} }} + \frac{{\partial^{2} v}}{{\partial y^{2} }}} \right) + a_{2} \left( {\frac{{\partial^{2} v}}{{\partial y^{2} }} + \frac{{\partial^{2} u}}{\partial x\partial y}} \right) \\ & \quad - a_{3} \left( {1 + \tau_{1} \omega_{1} \frac{\partial }{\partial t}} \right)\frac{\partial \theta }{{\partial y}} - a_{4} \left( {1 + \tau^{1} \omega_{1} \frac{\partial }{\partial t}} \right)\frac{\partial C}{{\partial y}} \\ & \quad + a_{5} \frac{{\partial q_{1} }}{\partial y} + a_{6} \frac{{\partial q_{2} }}{\partial y}, \\ \end{aligned}$$

17 $$\begin{aligned} \left( {\frac{{\partial^{2} \theta }}{{\partial x^{2} }} + \frac{{\partial^{2} \theta }}{{\partial y^{2} }}} \right) & = a_{7} \frac{\partial }{\partial t}\left( {1 + \tau_{0} \omega_{1} \frac{\partial }{\partial t}} \right)\theta + a_{8} \left( {\frac{{\partial^{2} u}}{\partial x\partial t} + \frac{{\partial^{2} v}}{\partial y\partial t}} \right) + a_{9} \frac{\partial }{\partial t}\left( {1 + \tau^{o} \omega_{1} \frac{\partial }{\partial t}} \right)C \\ & \quad + a_{10} \left( {1 + \tau_{o} \omega_{1} \frac{\partial }{\partial t}} \right)\frac{{\partial q_{1} }}{\partial t} \\ & \quad + a_{11} \left( {1 + \tau_{o} \omega_{1} \frac{\partial }{\partial t}} \right)\frac{{\partial q_{2} }}{\partial t}, \\ \end{aligned}$$

18 $$\begin{gathered} a_{12} \left( {\frac{{\partial^{2} q_{1} }}{{\partial x^{2} }} + \frac{{\partial^{2} q_{1} }}{{\partial y^{2} }}} \right) + a_{13} \left( {\frac{{\partial^{2} q_{2} }}{{\partial x^{2} }} + \frac{{\partial^{2} q_{2} }}{{\partial y^{2} }}} \right) - b\left( {\frac{\partial u}{{\partial x}} + \frac{\partial v}{{\partial y}}} \right) - a_{14} q_{1} - a_{15} q_{2} + a_{16} \theta + a_{17} C \hfill \\ = \alpha_{1} \left( {1 - a_{0} L_{N}^{2} \nabla^{2} } \right)\frac{{\partial^{2} q_{1} }}{{\partial t^{2} }}, \hfill \\ \end{gathered}$$

19 $$\begin{gathered} a_{13} \left( {\frac{{\partial^{2} q_{1} }}{{\partial x^{2} }} + \frac{{\partial^{2} q_{1} }}{{\partial y^{2} }}} \right) + a_{18} \left( {\frac{{\partial^{2} q_{2} }}{{\partial x^{2} }} + \frac{{\partial^{2} q_{2} }}{{\partial y^{2} }}} \right) - d\left( {\frac{\partial u}{{\partial x}} + \frac{\partial v}{{\partial y}}} \right) - a_{15} q_{1} - a_{19} q_{2} + a_{20} \theta + a_{21} C \hfill \\ = a_{22} \left( {1 - a_{0} L_{N}^{2} \nabla^{2} } \right)\frac{{\partial^{2} q_{2} }}{{\partial t^{2} }}, \hfill \\ \end{gathered}$$

20 $$\sigma_{xx} = a_{23} \frac{\partial u}{{\partial x}} + a_{24} \left( {\frac{\partial u}{{\partial x}} + \frac{\partial v}{{\partial y}}} \right) - \left( {1 + \tau_{1} \omega_{1} \frac{\partial }{\partial t}} \right)\theta - a_{25} \left( {1 + \tau^{1} \omega_{1} \frac{\partial }{\partial t}} \right)C + a_{26} q_{1} + a_{27} q_{2} ,$$

21 $$\sigma_{yy} = a_{23} \frac{\partial v}{{\partial y}} + a_{24} \left( {\frac{\partial u}{{\partial x}} + \frac{\partial v}{{\partial y}}} \right) - \left( {1 + \tau_{1} \omega_{1} \frac{\partial }{\partial t}} \right)\theta - a_{25} \left( {1 + \tau^{1} \omega_{1} \frac{\partial }{\partial t}} \right)C + a_{26} q_{1} + a_{27} q_{2} ,$$

22 $$\sigma_{xy} = a_{28} \left( {\frac{\partial u}{{\partial y}} + \frac{\partial v}{{\partial x}}} \right),$$

23 $$\sigma_{x} = a_{29} \frac{{\partial q_{1} }}{\partial x} + a_{30} \frac{{\partial q_{2} }}{\partial x},$$

24 $$\sigma_{y} = a_{29} \frac{{\partial q_{1} }}{\partial y} + a_{30} \frac{{\partial q_{2} }}{\partial y},$$

25 $$\chi_{x} = a_{31} \frac{{\partial q_{1} }}{\partial x} + a_{32} \frac{{\partial q_{2} }}{\partial x}$$

26 $$\chi_{y} = a_{31} \frac{{\partial q_{1} }}{\partial y} + a_{32} \frac{{\partial q_{2} }}{\partial y},$$

27 $$\begin{gathered} a_{33} \left( {1 + \tau^{1} \omega_{1} \frac{\partial }{\partial t}} \right)\left( {\frac{{\partial^{2} C}}{{\partial x^{2} }} + \frac{{\partial^{2} C}}{{\partial y^{2} }}} \right) = \left( {\frac{{\partial^{2} }}{{\partial x^{2} }} + \frac{{\partial^{2} }}{{\partial y^{2} }}} \right)\left( {\frac{\partial u}{{\partial x}} + \frac{\partial v}{{\partial y}}} \right) + a_{34} \left( {1 + \tau_{1} \omega_{1} \frac{\partial }{\partial t}} \right)\left( {\frac{{\partial^{2} \theta }}{{\partial x^{2} }} + \frac{{\partial^{2} \theta }}{{\partial y^{2} }}} \right) \hfill \\ + a_{35} \left( {\frac{{\partial^{2} q_{1} }}{{\partial x^{2} }} + \frac{{\partial^{2} q_{1} }}{{\partial y^{2} }}} \right) + a_{36} \left( {\frac{{\partial^{2} q_{2} }}{{\partial x^{2} }} + \frac{{\partial^{2} q_{2} }}{{\partial y^{2} }}} \right) + a_{37} \frac{\partial C}{{\partial t}} \hfill \\ \end{gathered}$$

where

$$a_{o} = \frac{{\omega_{1}^{2} }}{{ c_{1}^{2} }}$$, $$a_{1} = \frac{ \mu }{{ c_{1}^{2} }}$$, $$a_{2} = \frac{ \lambda + \mu }{{ c_{1}^{2} }}$$, $$a_{3} = \frac{{ \beta_{1} T_{o} }}{{c_{1}^{2} }}$$, $$a_{4} = \frac{{ \beta_{2} c_{o} }}{{c_{1}^{2} }}$$, $$a_{5} = \frac{{ b\alpha_{1} }}{{c_{1}^{2} k_{1} \omega_{1}^{2} }}$$, $$a_{6} = \frac{{ d\alpha_{1} }}{{c_{1}^{2} k_{1} \omega_{1}^{2} }}$$,$$a_{7} = \frac{{ \rho c_{e} c_{1}^{2} }}{{K\omega_{1} }}$$, $$a_{8} = \frac{{ \beta_{1} c_{1}^{2} }}{{K\omega_{1} }}$$, $$a_{9} = \frac{{ a_{c} c_{o} c_{1}^{2} }}{{K\omega_{1} }}$$, $$a_{10} = \frac{{\gamma_{1} \alpha_{1} c_{1}^{2} }}{{Kk_{1} \omega_{1}^{3} }}$$, $$a_{11} = \frac{{\gamma_{2} \alpha_{1} c_{1}^{2} }}{{Kk_{1} \omega_{1}^{3} }}$$, $$a_{12} = \frac{{\alpha \alpha_{1} }}{{k_{1} c_{1}^{2} }}$$, $$a_{13} = \frac{{b_{1} \alpha_{1} }}{{k_{1} c_{1}^{2} }}$$, $$a_{14} = \frac{{\alpha_{1}^{2} }}{{k_{1} \omega_{1}^{2} }}$$, $$a_{15} = \frac{{\alpha_{1} \alpha_{3} }}{{k_{1} \omega_{1}^{2} }}$$, $$a_{16} = \gamma_{1} T_{o}$$, $$a_{17} = \upsilon c_{o}$$, $$a_{18} = \frac{{\gamma \alpha_{1} }}{{k_{1} c_{1}^{2} }}$$, $$a_{19} = \frac{{\alpha_{1} \alpha_{2} }}{{k_{1} \omega_{1}^{2} }}$$, $$a_{20} = \gamma T_{o}$$, $$a_{21} = mc_{o}$$, $$a_{22} = \frac{{\alpha_{1} k_{2} }}{{k_{1} }}$$, $$a_{23} = \frac{ 2\mu }{{\beta_{1} T_{o} }}$$, $$a_{24} = \frac{ \lambda }{{\beta_{1} T_{o} }}$$, $$a_{25} = \frac{{ \beta_{2} c_{o} }}{{\beta_{1} T_{o} }}$$, $$a_{26} = \frac{{ b\alpha_{1} }}{{k_{1} \omega_{1}^{2} \beta_{1} T_{o} }}$$, $$a_{27} = \frac{{ d\alpha_{1} }}{{k_{1} \omega_{1}^{2} \beta_{1} T_{o} }}$$,$$a_{28} = \frac{ \mu }{{\beta_{1} T_{o} }}$$, $$a_{29} = \frac{{ \alpha_{1} }}{{k_{1} \omega_{1}^{2} }}$$, $$a_{30} = \frac{{ b_{1} \alpha_{1} }}{{k_{1} \omega_{1}^{2} }}$$,$$a_{31} = \frac{{ b_{1} \alpha_{1} }}{{\alpha k_{1} \omega_{1}^{2} }}$$, $$a_{32} = \frac{{ \gamma \alpha_{1} }}{{\alpha k_{1} \omega_{1}^{2} }}$$, $$a_{33} = \frac{{ b_{c} }}{{ \beta_{2} }}$$, $$a_{34} = \frac{{ a_{c} T_{o} }}{{ \beta_{2} }}$$, $$a_{35} = \frac{{ \upsilon \alpha_{1} }}{{k_{1} \omega_{1}^{2} \beta_{2} }}$$, $$a_{36} = \frac{{ m\alpha_{1} }}{{k_{1} \omega_{1}^{2} \beta_{2} }}$$, $$a_{37} = \frac{{c_{o} c_{1}^{2} }}{{\omega_{1} \beta_{2} D_{c} }}$$.

## Solution of the problem

In this section, we explore the utilization of Lame’s potential and normal mode methods to address the accuracy issue without imposing constraints on the field variables in the governing equations.

By Helmholtz’s theorem^26^, the displacement vector $\vec{u}$ can be written in the form:

28a $$\vec{u} = \underline {\nabla } {\Phi } + \underline {\nabla } \wedge {\vec{\Psi }},$$

where the two functions ${\Phi }$ and ${\vec{\Psi }}$ are known in the theory of elasticity, by Lame’s potentials representing irrotational and rotational parts of the displacement vector $\vec{u}$ respectively.

28 $$u = \frac{{\partial {\Phi }}}{\partial x} - \frac{{\partial {\Psi }}}{\partial y},v = \frac{{\partial {\Phi }}}{\partial y} + \frac{{\partial {\Psi }}}{\partial x},$$

29 $$\begin{gathered} \rho \frac{{\partial^{2} {\Phi }}}{{\partial t^{2} }}\left( {1 - a_{0} L_{N}^{2} \frac{{\partial^{2} {\Phi }}}{{\partial x^{2} }} - a_{0} L_{N}^{2} \frac{{\partial^{2} {\Phi }}}{{\partial y^{2} }} + a_{0} L_{N}^{2} \frac{{\partial^{2} {\Psi }}}{\partial x\partial y}} \right) - \rho {\Omega }^{2} {\Phi } + 2\rho {\Omega }\frac{{\partial {\Psi }}}{\partial t} = a_{38} \left( {\frac{{\partial^{2} {\Phi }}}{{\partial x^{2} }} + \frac{{\partial^{2} {\Phi }}}{{\partial y^{2} }}} \right) \hfill \\ - a_{1} \frac{{\partial^{2} {\Psi }}}{\partial x\partial y} - a_{3} \left( {1 + \tau_{1} \omega_{1} \frac{\partial }{\partial t}} \right)\theta - a_{4} \left( {1 + \tau^{1} \omega_{1} \frac{\partial }{\partial t}} \right)C + a_{5} q_{1} + a_{6} q_{2} , \hfill \\ \end{gathered}$$

30 $$\begin{gathered} \rho \frac{{\partial^{2} {\Psi }}}{{\partial t^{2} }} - \rho a_{0} L_{N}^{2} \frac{{\partial^{4} {\Phi }}}{{\partial x\partial y\partial t^{2} }} - \rho a_{0} L_{N}^{2} \frac{{\partial^{4} {\Psi }}}{{\partial x^{2} \partial t^{2} }} - \rho a_{0} L_{N}^{2} \frac{{\partial^{4} {\Psi }}}{{\partial y^{2} \partial t^{2} }} - \rho {\Omega }^{2} {\Psi } + 2\rho {\Omega }\frac{{\partial {\Phi }}}{\partial t} = a_{38} \frac{{\partial^{2} {\Phi }}}{\partial x\partial y} \hfill \\ + a_{1} \left( {\frac{{\partial^{2} {\Psi }}}{{\partial x^{2} }} + \frac{{\partial^{2} {\Psi }}}{{\partial y^{2} }}} \right), \hfill \\ \end{gathered}$$

31 $$\begin{aligned} \left( {\frac{{\partial^{2} \theta }}{{\partial x^{2} }} + \frac{{\partial^{2} \theta }}{{\partial y^{2} }}} \right) & = a_{7} \left( {1 + \tau_{0} \omega_{1} \frac{\partial }{\partial t}} \right)\frac{\partial \theta }{{\partial t}} + a_{8} \frac{{\partial^{3} {\Phi }}}{{\partial x^{2} \partial t}} + a_{8} \frac{{\partial^{3} {\Phi }}}{{\partial y^{2} \partial t}} + a_{9} \left( {1 + \tau^{o} \omega_{1} \frac{\partial }{\partial t}} \right)\frac{\partial C}{{\partial t}} \\ & \quad + a_{10} \left( {1 + \tau_{o} \omega_{1} \frac{\partial }{\partial t}} \right)\frac{{\partial q_{1} }}{\partial t} + a_{11} \left( {1 + \tau_{o} \omega_{1} \frac{\partial }{\partial t}} \right)\frac{{\partial q_{2} }}{\partial t}, \\ \end{aligned}$$

32 $$\begin{gathered} a_{12} \left( {\frac{{\partial^{2} q_{1} }}{{\partial x^{2} }} + \frac{{\partial^{2} q_{1} }}{{\partial y^{2} }}} \right) + a_{13} \left( {\frac{{\partial^{2} q_{2} }}{{\partial x^{2} }} + \frac{{\partial^{2} q_{2} }}{{\partial y^{2} }}} \right) - b\left( {\frac{{\partial^{2} {\Phi }}}{{\partial x^{2} }} + \frac{{\partial^{2} {\Phi }}}{{\partial y^{2} }}} \right) - a_{14} q_{1} - a_{15} q_{2} + a_{16} \theta + a_{17} C \hfill \\ = \alpha_{1} \left( {1 - a_{0} L_{N}^{2} \nabla^{2} } \right)\frac{{\partial^{2} q_{1} }}{{\partial t^{2} }}, \hfill \\ \end{gathered}$$

33 $$\begin{gathered} a_{13} \left( {\frac{{\partial^{2} q_{1} }}{{\partial x^{2} }} + \frac{{\partial^{2} q_{1} }}{{\partial y^{2} }}} \right) + a_{18} \left( {\frac{{\partial^{2} q_{2} }}{{\partial x^{2} }} + \frac{{\partial^{2} q_{2} }}{{\partial y^{2} }}} \right) - d\left( {\frac{{\partial^{2} {\Phi }}}{{\partial x^{2} }} + \frac{{\partial^{2} {\Phi }}}{{\partial y^{2} }}} \right) - a_{15} q_{1} - a_{19} q_{2} + a_{20} \theta + a_{21} C \hfill \\ = a_{22} \left( {1 - a_{0} L_{N}^{2} \nabla^{2} } \right)\frac{{\partial^{2} q_{2} }}{{\partial t^{2} }} \hfill \\ \end{gathered}$$

34 $$\begin{gathered} \sigma_{xx} = a_{23} \frac{{\partial^{2} {\Phi }}}{{\partial x^{2} }} + a_{24} \left( {\frac{{\partial^{2} {\Phi }}}{{\partial x^{2} }} + \frac{{\partial^{2} {\Phi }}}{{\partial y^{2} }}} \right) - a_{23} \frac{{\partial^{2} {\Psi }}}{\partial x\partial y} - \left( {1 + \tau_{1} \omega_{1} \frac{\partial }{\partial t}} \right)\theta - a_{25} \left( {1 + \tau^{1} \omega_{1} \frac{\partial }{\partial t}} \right)C \hfill \\ + a_{26} q_{1} + a_{27} q_{2} , \hfill \\ \end{gathered}$$

35 $$\begin{aligned} \sigma_{yy} & = a_{23} \frac{{\partial^{2} {\Phi }}}{{\partial y^{2} }} + a_{24} \left( {\frac{{\partial^{2} {\Phi }}}{{\partial x^{2} }} + \frac{{\partial^{2} {\Phi }}}{{\partial y^{2} }}} \right) + a_{23} \frac{{\partial^{2} {\Psi }}}{\partial x\partial y} - \left( {1 + \tau_{1} \omega_{1} \frac{\partial }{\partial t}} \right)\theta - a_{25} \left( {1 + \tau^{1} \omega_{1} \frac{\partial }{\partial t}} \right)C \\ & \quad + a_{26} q_{1} + a_{27} q_{2} , \\ \end{aligned}$$

36 $$\sigma_{xy} = a_{28} \left( {\frac{{\partial^{2} {\Psi }}}{{\partial x^{2} }} - \frac{{\partial^{2} {\Psi }}}{{\partial y^{2} }}} \right) + 2a_{28} \frac{{\partial^{2} {\Phi }}}{\partial x\partial y},$$

37 $$\sigma_{x} = a_{29} \frac{{\partial q_{1} }}{\partial x} + a_{30} \frac{{\partial q_{2} }}{\partial x},$$

38 $$\sigma_{y} = a_{29} \frac{{\partial q_{1} }}{\partial y} + a_{30} \frac{{\partial q_{2} }}{\partial y},$$

39 $$\chi_{x} = a_{31} \frac{{\partial q_{1} }}{\partial x} + a_{32} \frac{{\partial q_{2} }}{\partial x}$$

40 $$\chi_{y} = a_{31} \frac{{\partial q_{1} }}{\partial y} + a_{32} \frac{{\partial q_{2} }}{\partial y},$$

41 $$\begin{aligned} a_{33} \left( {1 + \tau^{1} \omega_{1} \frac{\partial }{\partial t}} \right)\left( {\frac{{\partial^{2} C}}{{\partial x^{2} }} + \frac{{\partial^{2} C}}{{\partial y^{2} }}} \right) & = \left( {\frac{{\partial^{4} {\Phi }}}{{\partial x^{4} }} + \frac{{\partial^{4} {\Phi }}}{{\partial y^{4} }}} \right) + a_{34} \left( {1 + \tau_{1} \omega_{1} \frac{\partial }{\partial t}} \right)\left( {\frac{{\partial^{2} \theta }}{{\partial x^{2} }} + \frac{{\partial^{2} \theta }}{{\partial y^{2} }}} \right) \\ & \quad + a_{35} \left( {\frac{{\partial^{2} q_{1} }}{{\partial x^{2} }} + \frac{{\partial^{2} q_{1} }}{{\partial y^{2} }}} \right) + a_{36} \left( {\frac{{\partial^{2} q_{2} }}{{\partial x^{2} }} + \frac{{\partial^{2} q_{2} }}{{\partial y^{2} }}} \right) + a_{37} \frac{\partial C}{{\partial t}} \\ \end{aligned}$$

The following normal modes can be used to investigate the physical variable solutions:

42 $$\left( {{\Phi },{\Psi },q_{1} , q_{2} ,\theta ,C,\sigma_{ij} } \right)\left( {x,y,t} \right) = \left( {{\Phi }^{*} ,{\Psi }^{*} ,q_{1}^{*} ,q_{2}^{*} ,\theta^{*} ,C^{*} ,\sigma_{ij}^{*} } \right)e^{{\left( {\omega t + iey} \right)}} .$$

where the angular frequency, the imaginary number, and the wave number in the z-direction are denoted by the letters $\omega , i,$ and e. Employing Eqs. (28) and (42), Eqs. (29–41) become, respectively:

43 $$(a_{39} - a_{41} D^{2} ){\Phi }^{*} + a_{42} \theta^{*} + a_{43} C^{*} - a_{5} q_{1}^{*} - a_{6} q_{2}^{*} = 0,$$

44 $$(a_{44} - a_{45} D^{2} ){\Psi }^{*} = 0,$$

45 $$(a_{46} D^{2} + a_{47} ){\Phi }^{*} - \left( {D^{2} - a_{48} } \right)\theta^{*} + a_{49} C^{*} + a_{50} q_{1}^{*} + a_{51} q_{2}^{*} = 0$$

46 $$(bD^{2} + e^{2} ){\Phi }^{*} - a_{16} \theta^{*} - a_{17} C^{*} - \left( {a_{52} D^{2} - a_{53} } \right)q_{1}^{*} - \left( {a_{13} D^{2} - a_{54} } \right)q_{2}^{*} = 0,$$

47 $$(e^{2} - dD^{2} ){\Phi }^{*} + a_{20} \theta^{*} + a_{21} C^{*} + \left( {a_{13} D^{2} - a_{55} } \right)q_{1}^{*} + \left( {a_{56} D^{2} - a_{57} } \right)q_{2}^{*} = 0,$$

48 $$(D^{4} + e^{4} ){\Phi }^{*} + a_{58} (D^{2} - e^{2} )\theta^{*} - a_{59} C^{*} + a_{35} \left( {D^{2} - e^{2} } \right)q_{1}^{*} + a_{36} \left( {D^{2} - e^{2} } \right)q_{2}^{*} = 0,$$

49 $$\begin{gathered} \sigma_{xx}^{*} = [a_{23} D^{2} + a_{24} \left( {D^{2} - e^{2} } \right)]{\Phi }^{*} - iea_{23} D{\Psi }^{*} - \left( {1 + \tau_{1} \omega_{1} \omega } \right)\theta^{*} - a_{25} \left( {1 + \tau^{1} \omega_{1} \omega } \right)C^{*} \hfill \\ + a_{26} q_{1}^{*} + a_{27} q_{2}^{*} \hfill \\ \end{gathered}$$

50 $$\begin{gathered} \sigma_{yy}^{*} = [ - a_{23} e^{2} + a_{24} \left( {D^{2} - e^{2} } \right)]{\Phi }^{*} + iea_{23} D{\Psi }^{*} - \left( {1 + \tau_{1} \omega_{1} \omega } \right)\theta^{*} - a_{25} \left( {1 + \tau^{1} \omega_{1} \omega } \right)C^{*} \hfill \\ + a_{26} q_{1}^{*} + a_{27} q_{2}^{*} , \hfill \\ \end{gathered}$$

51 $$\sigma_{xy}^{*} = a_{28} \left( {D^{2} + e^{2} } \right){\Psi }^{*} + 2iea_{28} D{\Phi }^{*} ,$$

52 $$\sigma_{x}^{*} = a_{29} Dq_{1}^{*} + a_{30} Dq_{2}^{*} ,$$

53 $$\sigma_{y}^{*} = iea_{29} q_{1}^{*} + iea_{30} q_{2}^{*} ,$$

54 $$\chi_{x}^{*} = a_{31} Dq_{1}^{*} + a_{32} Dq_{2}^{*} ,$$

55 $$\chi_{y}^{*} = iea_{31} q_{1}^{*} + iea_{32} q_{2}^{*} ,$$

where

$$D^{2} = \frac{{d^{2} }}{{dx^{2} }}$$, $$a_{38} = a_{1} + a_{2}$$, $$a_{39} = \rho \omega^{2} + \rho a_{o} L_{N}^{2} \omega^{2} e^{2} - \rho {\Omega }^{2} + a_{38} e^{2}$$, $$a_{40} = \rho a_{o} L_{N}^{2} \omega^{2}$$, $$a_{41} = a_{38} + a_{40}$$,$$a_{42} = a_{3} \left( {1 + \tau_{1} \omega_{1} \omega } \right)$$, $$a_{43} = a_{4} \left( {1 + \tau^{1} \omega_{1} \omega } \right)$$, $$a_{44} = \rho \omega^{2} + \rho a_{o} L_{N}^{2} \omega^{2} e^{2} - \rho {\Omega }^{2} + a_{1} e^{2}$$, $$a_{45} = \rho a_{o} L_{N}^{2} \omega^{2} + a_{1}$$,$$a_{46} = a_{8} \omega$$,$$a_{47} = a_{8} e^{2}$$,$$a_{48} = e^{2} + a_{7} \omega \left( {1 + \tau_{o} \omega_{1} \omega } \right)$$, $$a_{49} = a_{9} \omega \left( {1 + \tau^{o} \omega_{1} \omega } \right)$$, $$a_{50} = a_{10} \omega \left( {1 + \tau_{o} \omega_{1} \omega } \right)$$,$$a_{51} = a_{11} \omega \left( {1 + \tau_{o} \omega_{1} \omega } \right)$$, $$a_{52} = a_{12} + \alpha_{1} a_{o} L_{N}^{2} \omega^{2}$$,$$a_{53} = a_{14} + a_{12} e^{2} + \alpha_{1} \omega^{2} + \alpha_{1} a_{o} L_{N}^{2} \omega^{2} e^{2}$$,$$a_{54} = a_{13} e^{2} + a_{15}$$, $$a_{55} = e^{2} + a_{15}$$, $$a_{56} = a_{18} + a_{o} a_{22} L_{N}^{2} \omega^{2}$$, $$a_{57} = e^{2} + a_{19} + a_{22} \omega^{2} + a_{22} L_{N}^{2} \omega^{2} e^{2}$$, $$a_{58} = a_{34} \left( {1 + \tau_{1} \omega_{1} \omega } \right)$$,$$a_{59} = a_{33} \left( {1 + \tau^{1} \omega_{1} \omega } \right) - a_{37} \omega + a_{o} a_{37} L_{N}^{2} \omega$$.

Eliminating ${\Phi }^{*} \left( x \right),{ }\theta^{*} \left( x \right),q_{1}^{*} \left( x \right)$, $q_{2}^{*} \left( x \right)$ and $C^{*} \left( x \right)$ from Eqs. (43–47) yields the following tenth order.

$$\left[ {D^{10} + B_{11} D^{8} + B_{22} D^{6} + B_{33} D^{4} + B_{44} D^{2} + B_{55} } \right]\left\{ {{\Phi }^{*} \left( x \right),{ }\theta^{*} \left( x \right),C^{*} \left( x \right),q_{1}^{*} \left( x \right),{ }q_{2}^{*} \left( x \right)} \right\} = 0.                       $$

The characteristic equation of the last equation is

56 $$\lambda^{10} + B_{11} \lambda^{8} + B_{22} \lambda^{6} + B_{33} \lambda^{4} + B_{44} \lambda^{2} + B_{55} = 0.$$

where

The definitions for the involved coefficients $B_{11} ,B_{22} ,B_{33} ,B_{44}$, and $B_{55}$ are detailed in Appendix I, and $\lambda_{i} ,i = 1,2,3,4,5,6,7,8,9,10$ are the all roots for this equation.

The general solutions of Eqs. (43)–(47), bound as *x* → ∞, are given by:

57 $${\Phi }^{*} = \mathop \sum \limits_{i = 1}^{5} A_{i} e^{{ - \lambda_{i} x}} ,$$

58 $$q_{1}^{*} = \mathop \sum \limits_{i = 1}^{5} A_{i} H_{1i} e^{{ - \lambda_{i} x}} ,$$

59 $$q_{2}^{*} = \mathop \sum \limits_{i = 1}^{5} A_{i} H_{2i} e^{{ - \lambda_{i} x}} ,$$

60 $$\theta^{*} = \mathop \sum \limits_{i = 1}^{5} - A_{i} H_{3i} e^{{ - \lambda_{i} x}} ,$$

61 $$C^{*} = \mathop \sum \limits_{i = 1}^{5} A_{i} H_{4i} e^{{ - \lambda_{i} x}} ,$$

62 $${\Psi }^{*} = A_{6} e^{{ - \sqrt {\frac{{a_{44} }}{{a_{45} }}} x}} .$$

The displacement components may be calculated in the following manner by using Eqs. (57), (62) and (28):

63 $$u^{*} = \mathop \sum \limits_{i = 1}^{5} - \lambda_{i} A_{i} e^{{ - \lambda_{i} x}} - ieA_{6} e^{{ - \sqrt {\frac{{a_{44} }}{{a_{45} }}} x}} ,$$

64 $$v^{*} = \mathop \sum \limits_{i = 1}^{5} ieA_{i} e^{{ - \lambda_{i} x}} - \sqrt {\frac{{a_{44} }}{{a_{45} }}} A_{6} e^{{ - \sqrt {\frac{{\delta_{18} }}{{a_{12} }}} x}} ,$$

By using Eqs. (6)–(12), (28), and (22) the stress components may be determined

65 $$\begin{aligned} \sigma_{xx}^{*} & = \mathop \sum \limits_{i = 1}^{5} \left[ {a_{23} \lambda_{i}^{2} + a_{24} \left( {\lambda_{i}^{2} - e^{2} } \right) + \left( {1 + \tau_{1} \omega_{1} \omega } \right)H_{3i} - a_{25} \left( {1 + \tau^{1} \omega_{1} \omega } \right)H_{4i} + a_{26} H_{1i} + + a_{27} H_{2i} } \right]A_{i} e^{{ - \lambda_{i} x}} \\ & \quad + iea_{23} \sqrt {\frac{{a_{44} }}{{a_{45} }}} A_{6} e^{{ - \sqrt {\frac{{a_{44} }}{{a_{45} }}} x}} , \\ \end{aligned}$$

66 $$\begin{aligned} \sigma_{yy}^{*} & = \mathop \sum \limits_{i = 1}^{5} \left[ { - a_{23} e^{2} + a_{24} \left( {\lambda_{i}^{2} - e^{2} } \right) + \left( {1 + \tau_{1} \omega_{1} \omega } \right)H_{3i} - a_{25} \left( {1 + \tau^{1} \omega_{1} \omega } \right)H_{4i} + a_{26} H_{1i} + + a_{27} H_{2i} } \right]A_{i} e^{{ - \lambda_{i} x}} \\ & \quad - iea_{23} \sqrt {\frac{{a_{44} }}{{a_{45} }}} A_{6} e^{{ - \sqrt {\frac{{a_{44} }}{{a_{45} }}} x}} , \\ \end{aligned}$$

67 $$\sigma_{xy}^{*} = a_{28} \left( {\frac{{a_{44} }}{{a_{45} }} + e^{2} } \right)A_{6} e^{{ - \sqrt {\frac{{a_{44} }}{{a_{45} }}} x}} - 2\mathop \sum \limits_{i = 1}^{5} iea_{28} \lambda_{i} A_{i} e^{{ - \lambda_{i} x}} ,$$

68 $$\sigma_{x}^{*} = - \mathop \sum \limits_{i = 1}^{5} \left[ {a_{29} H_{1i} + a_{30} H_{2i} } \right]\lambda_{i} A_{i} e^{{ - \lambda_{i} x}} ,$$

69 $$\sigma_{y}^{*} = \mathop \sum \limits_{i = 1}^{5} [iea_{29} H_{1i} + iea_{30} H_{2i} ]A_{i} e^{{ - \lambda_{i} x}} ,$$

70 $$\chi_{x}^{*} = \mathop \sum \limits_{i = 1}^{5} [ - a_{31} \lambda_{i} H_{1i} - a_{32} \lambda_{i} H_{2i} ]A_{i} e^{{ - \lambda_{i} x}} ,$$

71 $$\chi_{y}^{*} = \mathop \sum \limits_{i = 1}^{5} \left[ {iea_{31} H_{1i} + iea_{32} H_{2i} } \right]A_{i} e^{{ - \lambda_{i} x}} .$$

where

The definitions for the coefficients $H_{1i} ,H_{2i} ,H_{3i} ,H_{4i} ,\left( {i = 1,2,3,4,5} \right)$ involved are detailed in Appendix II.

## Boundary conditions

In this section, we determine the values of the unspecified parameters (${A}_{i}$) by making specific assumptions regarding the boundary conditions at the free surface when (*x* = *0*).

The normal stress boundary condition is

72 $$\sigma_{xx} = 0$$

Vanishing of equilibrated stress corresponding to pores,

73 $$\sigma_{x} = 0,$$

Vanishing of equilibrated stress corresponding to fissures,

74 $$\chi_{x} = 0$$

The thermal load boundary condition is

75 $$\theta^{*} = P$$

The shearing stress boundary condition is

76 $$\sigma_{xy} = 0$$

Vanishing of diffusion, yields

77 $$C = 0$$

Combining Eqs. (47–51) yields six Eqs. for the constants $A_{1}$, $A_{2}$,$A_{3}$, $A_{4}$,$A_{5}$ and $A_{6}$.

78 $$\begin{gathered} s_{1} A_{1} + s_{2} A_{2} + s_{3} A_{3} + s_{4} A_{4} + s_{5} A_{5} + s_{6} A_{6} = 0, \hfill \\ H_{31} A_{1} + H_{32} A_{2} + H_{33} A_{3} + H_{34} A_{4} + H_{35} A_{5} = P, \hfill \\ H_{41} A_{1} + H_{42} A_{2} + H_{43} A_{3} + H_{44} A_{4} + H_{45} A_{5} = 0, \hfill \\ s_{7} A_{1} + s_{8} A_{2} + s_{9} A_{3} + s_{10} A_{4} + s_{11} A_{5} - s_{12} A_{6} = 0, \hfill \\ s_{13} A_{1} + s_{14} A_{2} + s_{15} A_{3} + s_{16} A_{4} + s_{17} A_{5} = 0, \hfill \\ s_{18} A_{1} + s_{19} A_{2} + s_{20} A_{3} + s_{21} A_{4} + s_{22} A_{5} = 0, \hfill \\ \end{gathered}$$

where the definitions for the coefficients P and $s_{i} ,\left( {i = 1,2,3, \ldots 22} \right)$ involved are detailed in Appendix III. To calculate the constants $A_{1}$, $A_{2}$,$A_{3}$, $A_{4}$,$A_{5}$ and $A_{6}$ Cramer’s method is applied on Eqs. (78).

79 $$A_{1} = \frac{{\Delta A_{1} }}{\Delta },A_{2} = \frac{{\Delta A_{2} }}{\Delta },A_{3} = \frac{{\Delta A_{3} }}{\Delta },A_{4} = \frac{{\Delta A_{4} }}{\Delta },A_{5} = \frac{{\Delta A_{5} }}{\Delta },A_{6} = \frac{{\Delta A_{6} }}{\Delta }.$$

The following dimensionless expressions of physical quantities ($\theta$, $q_{1}$,$q_{2}$, $u$, $v$,$\sigma_{xx}$,$\sigma_{yy}$, $\sigma_{xy}$) can be derived from Eqs. (57)–(71) and (42)

80 $${\Phi }\left( {x,y,t} \right) = \left\{ {\mathop \sum \limits_{i = 1}^{5} A_{i} e^{{ - \lambda_{i} x}} } \right\}e^{{\left( {\omega t + iey} \right)}} ,$$

81 $$q_{1} \left( {x,y,t} \right) = \left\{ {\mathop \sum \limits_{i = 1}^{5} A_{i} H_{1i} e^{{ - \lambda_{i} x}} } \right\}e^{{\left( {\omega t + iey} \right)}} ,$$

82 $$q_{2} \left( {x,y,t} \right) = \left\{ {\mathop \sum \limits_{i = 1}^{5} A_{i} H_{2i} e^{{ - \lambda_{i} x}} } \right\}e^{{\left( {\omega t + iey} \right)}} ,$$

83 $$\theta \left( {x,y,t} \right) = \left\{ {\mathop \sum \limits_{i = 1}^{5} - A_{i} H_{3i} e^{{ - \lambda_{i} x}} } \right\}e^{{\left( {\omega t + iey} \right)}} ,$$

84 $$C\left( {x,y,t} \right) = \left\{ {\mathop \sum \limits_{i = 1}^{5} A_{i} H_{4i} e^{{ - \lambda_{i} x}} } \right\}e^{{\left( {\omega t + iey} \right)}} ,$$

85 $${\Psi }\left( {x,y,t} \right) = \left\{ {A_{6} e^{{ - \sqrt {\frac{{a_{44} }}{{a_{45} }}} x}} } \right\}e^{{\left( {\omega t + iey} \right)}} .$$

88 $$u\left( {x,y,t} \right) = \{ \mathop \sum \limits_{i = 1}^{5} - \lambda_{i} A_{i} e^{{ - \lambda_{i} x}} - ieA_{6} e^{{ - \sqrt {\frac{{a_{44} }}{{a_{45} }}} x}} \} e^{{\left( {\omega t + iey} \right)}} ,$$

87 $$v\left( {x,y,t} \right) = \left\{ {\mathop \sum \limits_{i = 1}^{5} ieA_{i} e^{{ - \lambda_{i} x}} - \sqrt {\frac{{a_{44} }}{{a_{45} }}} A_{6} e^{{ - \sqrt {\frac{{\delta_{18} }}{{a_{12} }}} x}} } \right\}e^{{\left( {\omega t + iey} \right)}} ,$$

88 $$\begin{gathered} \sigma_{xx} \left( {x,y,t} \right) = \left\{ {\mathop \sum \limits_{i = 1}^{5} \left[ {a_{23} \lambda_{i}^{2} + a_{24} \left( {\lambda_{i}^{2} - e^{2} } \right) + \left( {1 + \tau_{1} \omega_{1} \omega } \right)H_{3i} - a_{25} \left( {1 + \tau^{1} \omega_{1} \omega } \right)H_{4i} + a_{26} H_{1i} + a_{27} H_{2i} } \right]} \right. \hfill \\ \left. {A_{i} e^{{ - \lambda_{i} x}} + iea_{23} \sqrt {\frac{{a_{44} }}{{a_{45} }}} A_{6} e^{{ - \sqrt {\frac{{a_{44} }}{{a_{45} }}} x}} } \right\}e^{{\left( {\omega t + iey} \right)}} , \hfill \\ \end{gathered}$$

89 $$\begin{gathered} \sigma_{yy} \left( {x,y,t} \right) = \left\{ {\mathop \sum \limits_{i = 1}^{5} \left[ { - a_{23} e^{2} + a_{24} \left( {\lambda_{i}^{2} - e^{2} } \right) + \left( {1 + \tau_{1} \omega_{1} \omega } \right)H_{3i} - a_{25} \left( {1 + \tau^{1} \omega_{1} \omega } \right)H_{4i} + a_{26} H_{1i} + a_{27} H_{2i} } \right]A_{i} e^{{ - \lambda_{i} x}} } \right. \hfill \\ \left. { - iea_{23} \sqrt {\frac{{a_{44} }}{{a_{45} }}} A_{6} e^{{ - \sqrt {\frac{{a_{44} }}{{a_{45} }}} x}} \} e^{{\left( {\omega t + iey} \right)}} } \right\}, \hfill \\ \end{gathered}$$

90 $$\sigma_{xy} \left( {x,y,t} \right) = \left\{ {a_{28} \left( {\frac{{a_{44} }}{{a_{45} }} + e^{2} } \right)A_{6} e^{{ - \sqrt {\frac{{a_{44} }}{{a_{45} }}} x}} - 2\mathop \sum \limits_{i = 1}^{5} iea_{28} \lambda_{i} A_{i} e^{{ - \lambda_{i} x}} } \right\}e^{{\left( {\omega t + iey} \right)}} ,$$

91 $$\sigma_{x} \left( {x,y,t} \right) = \left\{ { - \mathop \sum \limits_{i = 1}^{5} \left[ {a_{29} H_{1i} + a_{30} H_{2i} } \right]\lambda_{i} A_{i} e^{{ - \lambda_{i} x}} } \right\}e^{{\left( {\omega t + iey} \right)}} ,$$

92 $$\sigma_{y} \left( {x,y,t} \right) = \left\{ {\mathop \sum \limits_{i = 1}^{5} [iea_{29} H_{1i} + iea_{30} H_{2i} ]A_{i} e^{{ - \lambda_{i} x}} } \right\}e^{{\left( {\omega t + iey} \right)}} ,$$

93 $$\chi_{x} \left( {x,y,t} \right) = \left\{ {\mathop \sum \limits_{i = 1}^{5} [ - a_{31} \lambda_{i} H_{1i} - a_{32} \lambda_{i} H_{2i} ]A_{i} e^{{ - \lambda_{i} x}} } \right\}e^{{\left( {\omega t + iey} \right)}} ,$$

94 $$\chi_{y} \left( {x,y,t} \right) = \left\{ {\mathop \sum \limits_{i = 1}^{5} \left[ {iea_{31} H_{1i} + iea_{32} H_{2i} } \right]A_{i} e^{{ - \lambda_{i} x}} } \right\}e^{{\left( {\omega t + iey} \right)}} .$$

## Numerical results and discussion

This section focuses on numerical computation, presenting simulations for nonlocal thermoelastic half-space with double porosity, considering rotation effects. MATLAB software is utilized to obtain numerical results, examining key fields such as temperature, diffusion, displacement, equilibrated stress, and thermal stress. The necessary data for modelling a thermoelastic half-space with double porosity under rotation influence are taken from Sherief and Saleh^27^, utilizing copper material’s physical constants (Table 1) expressed in SI units.

**Table 1:** Numerical values of the material constants.

Unit	Symbol	Value	Unit	Symbol	Value
N m^−2^	$\lambda$	$7.76 \times 10^{10}$	N m^−2^	$\gamma_{1}$	$0.16 \times 10^{5}$
N m^−2^	$\mu$	$3.86 \times 10^{10}$	N m^−2^	$\gamma_{2}$	$0.219 \times 10^{5}$
kg m^−3^	$\rho$	8954	N m^−2^	$b$	$0.9 \times 10^{10}$
K	$T_{o}$	293	N	$b_{1}$	$0.12 \times 10^{ - 5}$
Sec(s)	$\tau_{o}$	0.02	m^5^ Kg^−1^ s^−2^	$b_{c}$	$0.9 \times 10^{4}$
Sec(s)	$\tau^{o}$	$0.2$	N m^−2^	$d$	$0.1 \times 10^{10}$
Sec(s)	$\tau_{1}$	$0.03$	N m^−2^ s^2^	$k_{1}$	$0.1456 \times 10^{ - 12}$
Sec(s)	$\tau^{1}$	$0.3$	N m^−2^ s^2^	$k_{2}$	$0.1546 \times 10^{ - 12}$
N	$\alpha$	$1.3 \times 10^{ - 5}$	kg s m^−3^	$D_{c}$	$0.85 \times 10^{ - 8}$
N m^−2^	$\alpha_{1}$	$2.3 \times 10^{10}$	N	$\upsilon$	$2.9 \times 10^{12}$
N m^−2^	$\alpha_{2}$	$2.4 \times 10^{10}$	W m^−1^ K^−1^	$K$	$386$
N m^−2^	$\alpha_{3}$	$2.5 \times 10^{10}$	N	$m$	$2.9 \times 10^{10}$
K^−1^	$\alpha_{t}$	$1.78 \times 10^{ - 5}$	m^−2^ s^2^ K^−1^	$C_{e}$	$3831$
m^3^ Kg^−1^	$\alpha_{c}$	$1.2 \times 10^{ - 4}$	Sec(s)	$t$	$0.1$
N	$\gamma$	$1.1 \times 10^{ - 5}$	m^−2^ K^−1^ s^−2^	$a_{c}$	$1.2 \times 10^{4}$

**Fig. 2:**
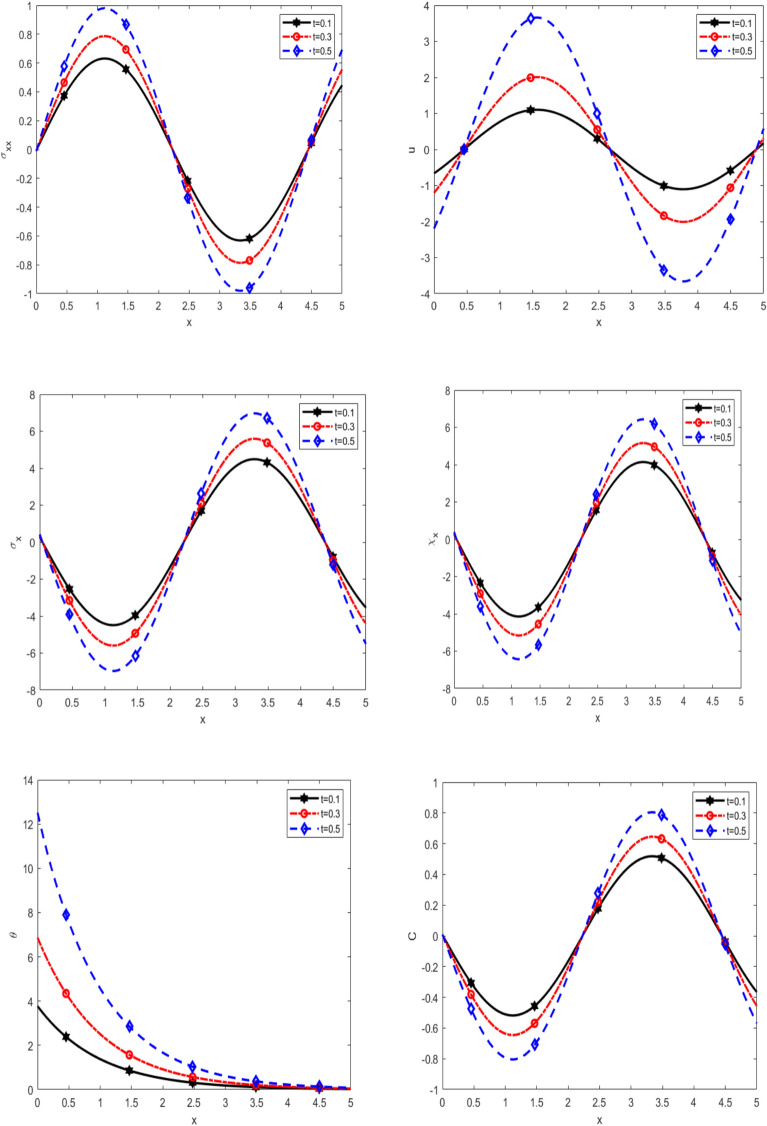
Effect of time on the main physical quantities versus* x*.

Figure 2 depicts the variation of normal stress $$\sigma_{xx}$$, displacement $$u$$, equilibrated stress $$\sigma_{x} ,\chi_{x}$$, temperature $$\theta$$, and diffusion $$C$$ with respect to $$x -$$ axis for different time values ($$0.1, 0.3$$ and $$0.5$$) under thermal load. It is noticed that $$\sigma_{xx}$$ increases with time ($$t)$$ in the interval $$0 \le x \le 2.5$$, decreases with time ($$t)$$ in the interval $$2.5 \le x \le 4.5,$$ and increases again in the interval $$4.5 \le x \le 5$$.Furthermore, the displacement (u) changes with time, dropping initially (0 ≤ x ≤ 0.5), rising (0.5 ≤ x ≤ 3), and then falling (3 ≤ x ≤ 5). In addition to the quantities $$\sigma_{x} ,\chi_{x}$$ and C exhibit a non-uniform temporal dependence, decreasing in the interval 0 ≤ x ≤ 2.5, increasing between 2.5 ≤ x ≤ 4.5, and decreasing again between 4.5 ≤ x ≤ 5. It is clear that the temperature distribution exhibits a steady rise to a peak at a specific distance, followed by a sharp decline, ultimately approaching zero. Across all scenarios, temperature trends converge to zero as distance increases. It increases proportionally with time. Initially, $$\sigma_{xx}$$ exhibits vibrational behaviour, shifting from positive to negative, whereas $$\sigma_{x} ,\chi_{x}$$, and C shift from negative to positive. The results underscore the substantial impact of time on stress distribution, displacement, diffusion, and temperature evolution. The observed oscillatory trends reveal a dynamic interplay between mechanical and thermal effects, highlighting the importance of time-dependent analysis in evaluating material behaviour under thermal loads. Recognizing these transient effects is essential for designing materials and structures that withstand thermal fluctuations, ensuring long-term stability and performance.

**Fig. 3:**
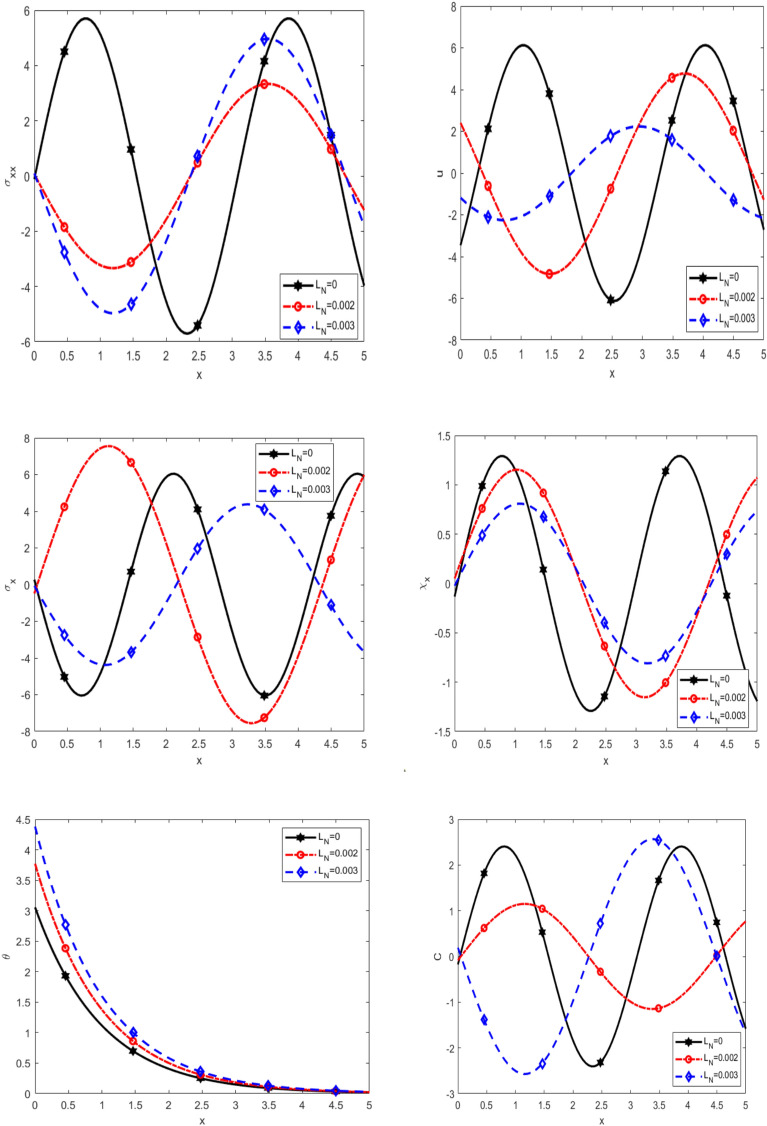
Effect of nonlocal on the main physical quantities versus* x*.

Figure 3 illustrates the variation of $\sigma_{xx}$, *u*, $\sigma_{x}$, $\chi_{x}$, θ, and C with respect to the $x$-axis for different nonlocal parameter values ($L_{n}$ = 0, 0.002, 0.003) under thermal load. We observe that $\sigma_{xx}$ decreases in the interval (0 ≤ x ≤ 1.5), increases in (1.5 ≤ x ≤ 2.5), decreases (2.5 ≤ x ≤ 4.5), and increases again in (4.5 ≤ x ≤ 5) with increasing $L_{n}$. Also, the displacement (u) decreases in (0 ≤ x ≤ 0.5), increases in (0.5 ≤ x ≤ 2.5), and decreases in (2.5 ≤ x ≤ 5) with $L_{n}$. As well as, $\sigma_{x}$ and $\chi_{x}$ decrease in (0 ≤ x ≤ 2.5), increase in (2.5 ≤ x ≤ 4.5), and decrease in (4.5 ≤ x ≤ 5) with $L_{n}$. Diffusion (C) increases in (0 ≤ x ≤ 2.5), decreases in (2.5 ≤ x ≤ 4.5), and increases in (4.5 ≤ x ≤ 5) with $L_{n}$. Temperature (θ) peaks and declines to zero. It trends converge to zero as distance increases and decreases with increasing $L_{n}$. Overall, these results highlight the significant influence of nonlocality on mechanical and thermal responses. The alternating trends in stress, displacement, and diffusion emphasize the complex interplay between nonlocal elasticity and material behavior under thermal loading. Understanding these effects is crucial for designing materials and structures where nonlocal interactions play a dominant role, such as in nano-scale materials, porous media, and advanced composites.

**Fig. 4:**
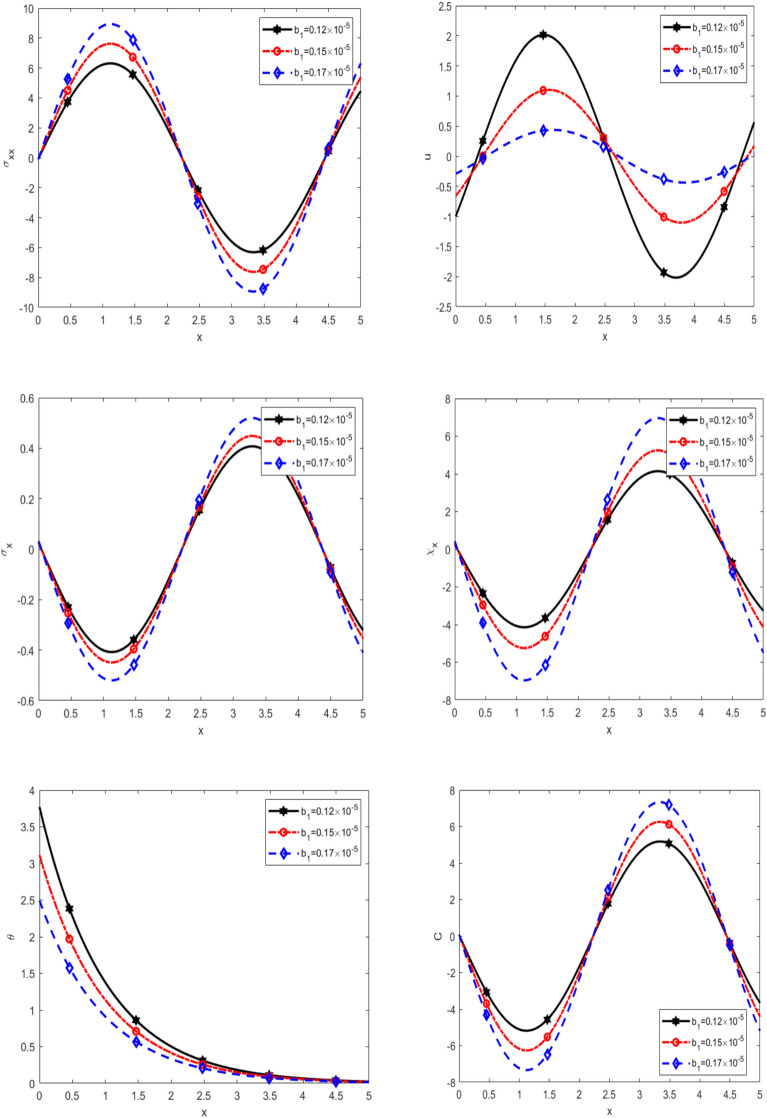
Effect of double porosity parameter due to pores on the physical quantities with *x*.

Figure 4 introduces the variation of normal stress $\sigma_{xx}$, displacement $u$, equilibrated stress $\sigma_{x} ,\chi_{x}$, temperature $\theta$, and diffusion $C$ with respect to $x$-axis for different values of porosity due to pores $b_{1}$ namely ($0.12 \times 10^{ - 5} ,0.15 \times 10^{ - 5}$ and $0.17 \times 10^{ - 5}$) under thermal load. It is noticed that $\sigma_{xx}$ is increasing with increasing $b_{1}$ in the interval $0 \le x \le 2.5$, while it decreases with increasing $b_{1}$ in the interval $2.5 \le x \le 4.5,$ as well it increases with increasing $b_{1}$ in the interval $4.5 \le x \le 5,$ $u$ is increasing with increasing $b_{1}$ in the interval $0 \le x \le 0.5$, while it decreases with increasing $b_{1}$ in the interval $0.5 \le x \le 2.5,$ as well it increases with increasing $b_{1}$ in the interval $2.5 \le x \le 4.5,$ furthermore it decreases with increasing $b_{1}$ in the interval $4.5 \le x \le 5,$ in addition to $\sigma_{x} ,\chi_{x} ,C$ are decreasing in the interval $0 \le x \le 2.5,$ while they are increasing with increasing $b_{1}$ in the interval $2.5 \le x \le 4.5,$ as well they are decreases with increasing $b_{1}$ in the interval $4.5 \le x \le 5,$ on the other hand, $\theta$ rises steadily to a peak at a certain distance, after which it experiences a sharp decline, ultimately approaching zero. In all scenarios, the temperature trends converge to zero as the distance increases. It is observed that the temperature decreases with increasing of porosity. It is noted that the $\sigma_{xx}$ initially, the behaviour of vibrations and it shift from positive to negative, while that the rest of the quantities *u*, $\sigma_{x} ,\chi_{x} ,C$ behaviour of vibrations in the range $x$-axis. The temperature distribution* θ* follows a distinct trend, rising to a peak at a certain distance before experiencing a sharp decline and ultimately converging to zero as *x* increases. This behavior indicates a localized thermal accumulation followed by dissipation. Moreover, the temperature decreases with increasing porosity, suggesting that higher porosity enhances thermal conduction or diffusion, leading to more efficient heat dissipation. Finally, the vibrational characteristics of the system are notable. The normal stress $\sigma_{xx}$ exhibits an alternating pattern, shifting from positive to negative, indicative of wave-like stress propagation due to thermal and mechanical interactions. The remaining quantities (*u*, $\sigma_{x} ,\chi_{x}$, and *C*) also display oscillatory behavior along the *x*-axis, reinforcing the presence of vibrational effects induced by porosity and thermal loading. These observations highlight the intricate coupling between porosity, stress distribution, displacement, temperature, and diffusion, underscoring the necessity of considering porosity effects in thermal and mechanical analysis.

**Fig. 5:**
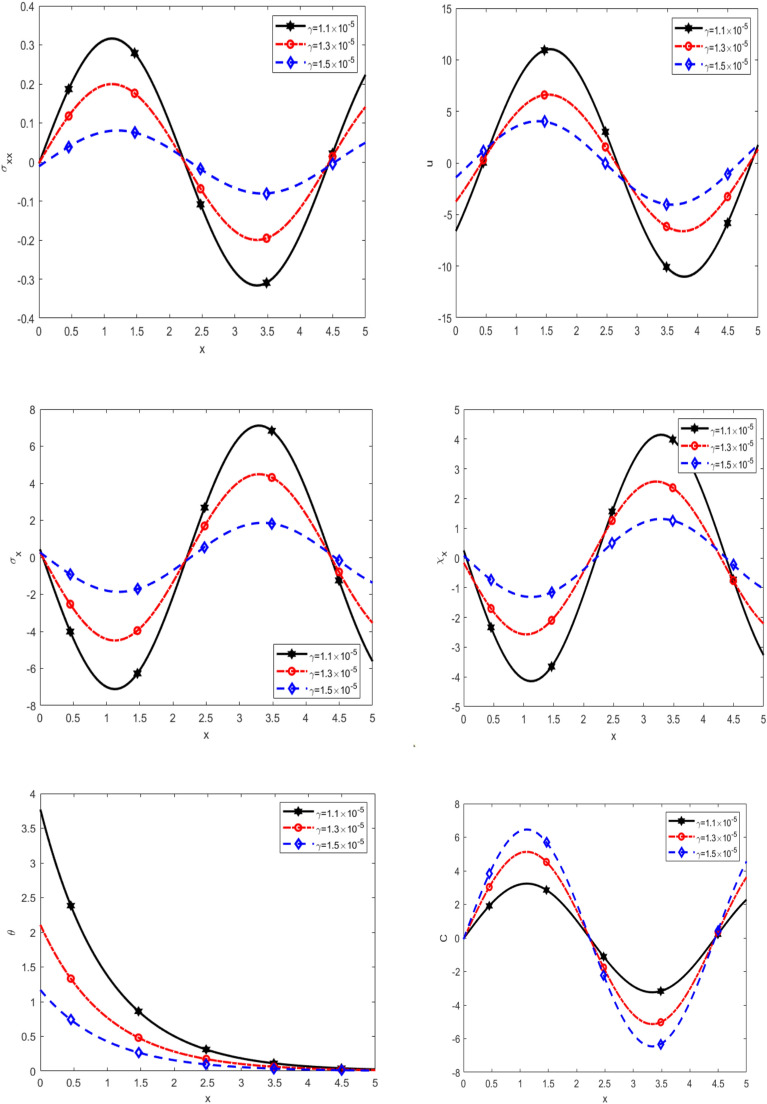
Effect of double porosity parameter due to fissures on the physical quantities.

Figure 5 presents the variation of normal stress $\sigma_{xx}$, displacement $u$, equilibrated stress $\sigma_{x} ,\chi_{x}$, temperature $\theta$, and diffusion $C$ with respect to $x$-axis for different values of porosity due to fissures $\gamma$ namely ($1.1x10^{ - 5} ,1.3x10^{ - 5}$ and $1.5x10^{ - 5}$) under thermal load. It is noticed that $\sigma_{xx}$ is decreasing with increasing $\gamma$ in the interval $0 \le x \le 2.5$, while it increases with increasing $\gamma$ in the interval $2.5 \le x \le 4.5,$ as well it decreases with increasing $\gamma$ in the interval $4.5 \le x \le 5,$ $u$ is increasing with increasing $\gamma$ in the interval $0 \le x \le 0.5$, while it decreases with increasing $\gamma$ in the interval $0.5 \le x \le 3,$ as well it increases with increasing $\gamma$ in the interval $3 \le x \le 5,$ in addition to $\sigma_{x} ,\chi_{x} ,C$ are decreasing with increasing $\gamma$ in the interval $0 \le x \le 2.5,$ while they are increasing with increasing $\gamma$ in the interval $2.5 \le x \le 4.5,$ as well they are decreases with increasing $\gamma$ in the interval $4.5 \le x \le 5,$ on the other hand, $\theta$ rises steadily to a peak at a certain distance, after which it experiences a sharp decline, ultimately approaching zero. In all scenarios, the temperature trends converge to zero as the distance increases. It is observed that the temperature increases with increasing of rotation. It is noted that the $\sigma_{xx}$ initially, the behaviour of vibrations and it shift from positive to negative, while that the rest of the quantities *u*, $\sigma_{x} ,\chi_{x} ,C$ behaviour of vibrations in the range $x$-axis. This refined explanation provides a clearer and more insightful interpretation of the physical and mechanical significance of the observed trends.

**Fig. 6:**
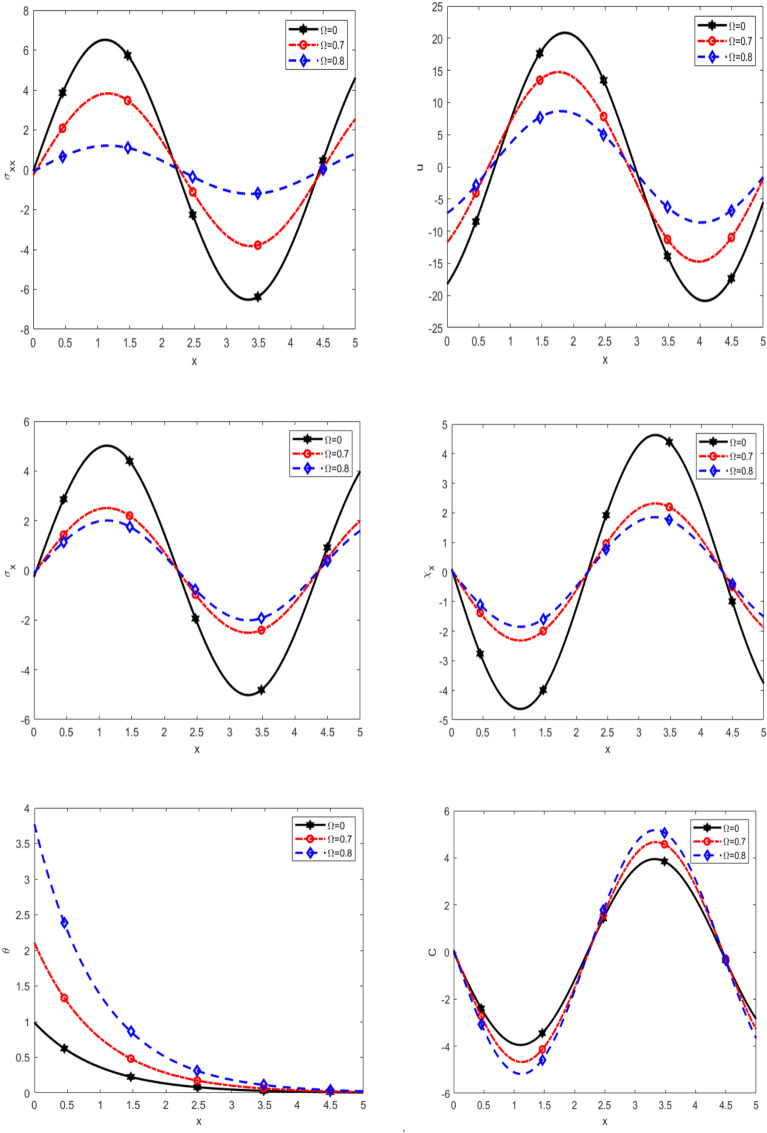
Effect of rotation on the main physical quantities versus* x*.

Figure 6 reveals the variation of normal stress $$\sigma_{xx}$$, displacement $$u$$, equilibrated stress $$\sigma_{x} ,\chi_{x}$$, temperature $$\theta$$, and diffusion $$C$$ with respect to $$x$$-axis for different values of rotation $$\Omega$$ namely $$(0, 0.7$$ and $$0.8)$$ for thermal load. It is noticed that $$\sigma_{xx}$$ is decreasing with increasing $$\Omega$$ in the interval $$0 \le x \le 2.5$$, while it decreases with increasing $$\Omega$$ in the interval $$2.5 \le x \le 4.5,$$ as well it increases with increasing $$\Omega$$ in the interval $$4.5 \le x \le 5,$$ $$u$$ is increasing with increasing $$\Omega$$ in the interval $$0 \le x \le 0.5$$, while it decreases with increasing $$\Omega$$ in the interval $$0.5 \le x \le 3,$$ as well it increases with increasing $$\Omega$$ in the interval $$3 \le x \le 5,$$ in addition to $$\sigma_{x} ,\chi_{x} ,C$$ are decreasing with increasing $$\Omega$$ in the interval $$0 \le x \le 2.5,$$ while they are decreasing with increasing $$\Omega$$ in the interval $$2.5 \le x \le 4.5,$$ as well they are increases with increasing $$\Omega$$ in the interval $$4.5 \le x \le 5,$$ on the other hand, $$\theta$$ rises steadily to a peak at a certain distance, after which it experiences a sharp decline, ultimately approaching zero. In all scenarios, the temperature trends converge to zero as the distance increases. It is observed that the temperature decreases with increasing of nonlocal parameter. It is noted that the $$\sigma_{xx}$$ initially, the behaviour of vibrations and it shift from positive to negative, while that the rest of the quantities *u*, $$\sigma_{x} ,\chi_{x} ,C$$ behaviour of vibrations in the range $$x$$-axis. These results provide deeper insight into the mechanical and thermal responses of the system, demonstrating how rotation and nonlocal effects contribute to stress redistribution, displacement modulation, and thermal diffusion. Understanding these interactions is crucial for optimizing material performance in applications involving rotational and nonlocal phenomena.

**Fig. 7:**
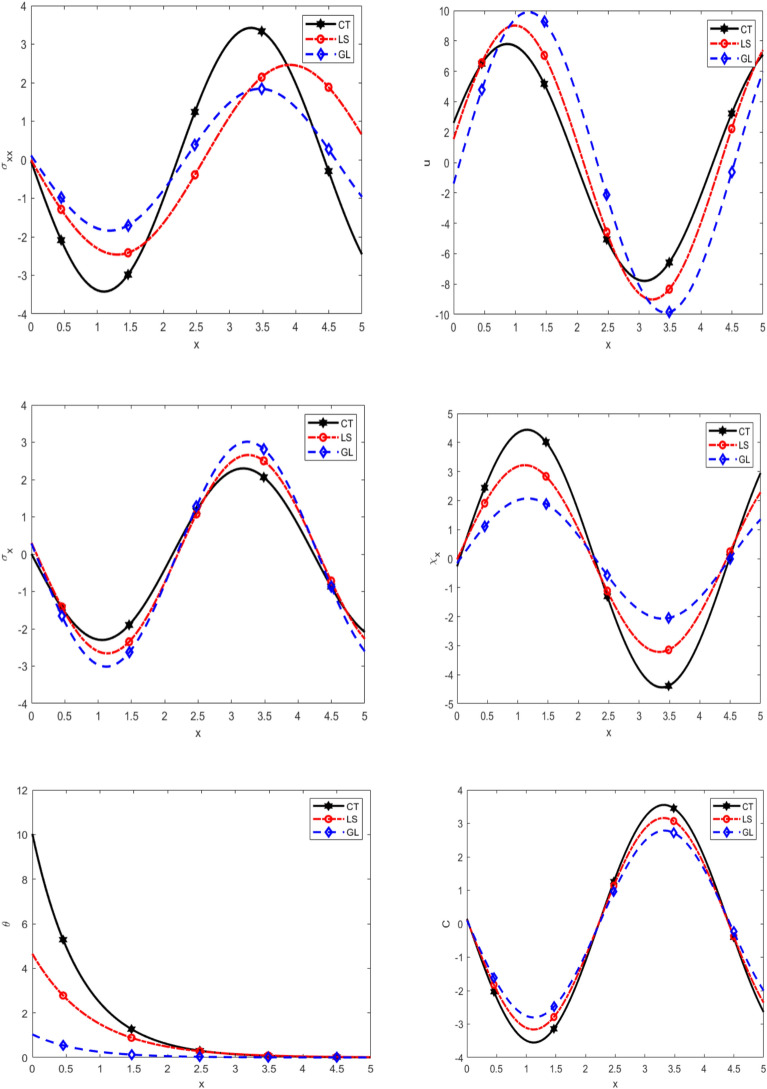
Comparison of main physical quantities with respect to *x* at different theories.

Figure 7 examines the variation of normal stress $\sigma_{xx}$, displacement $u$, equilibrated stress $\sigma_{x} ,\chi_{x}$, temperature $\theta$, and diffusion $C$ with respect to $x -$ axis for different theories under thermal load. At first, it is observed that the values of $\sigma_{xx} ,u,C$ increase in the range $0 \prec x \prec 2.5$ to theories $GL \succ LS \succ CT$ and decreases in the range $2.5 \prec x \prec 5$ to theories $CT \succ LS \succ GL$, while the values of $\sigma_{x} ,\chi_{x} ,$ decrease in the range $0 \prec x \prec 2.5$ to theories $CT \succ LS \succ GL$ and increase in the range $2.5 \prec x \prec 5$ to theories $GL \succ LS \succ CT$. Also, it is observed that the values of ${\uptheta }$ decrease in the range $0 \prec x \prec 5$ to $GL \prec LS \prec CT$ theories. This behavior can be attributed to the differences in the underlying assumptions of each theory regarding thermal and mechanical coupling effects. Specifically, the CT theory accounts for enhanced thermal diffusion and mechanical interactions, leading to higher stress and diffusion values near the heated region. In contrast, the GL theory exhibits a more gradual response, likely due to its more restrictive assumptions on stress and displacement distribution. The inverse behavior of $\sigma_{x} ,\chi_{x}$ compared to $\sigma_{xx} ,u$ and $C$ suggests a redistribution of internal forces to maintain equilibrium under thermal loading, which varies according to the theoretical framework considered.

## Conclusion

The primary aim of this research is to develop a mathematical model that characterizes the behaviour of normal stress, normal displacement, equilibrated stresses, temperature, and diffusion under nonlocal and thermal load in an infinite rotating generalized thermoelastic medium with diffusion and double porosity, within the framework of G–L theory. We utilize the normal mode analysis technique, which offers the advantage of finding exact solutions without imposing any assumptions on the field variables. Subsequently, we consider a copper-like material for numerical computation, and theoretical predictions are illustrated through various figures. The theoretical and numerical results indicate that factors such as nonlocal, time, double porosity parameter, rotation, and diffusion significantly influence the studied field variables. Additionally, comparisons have been made among CT, LS, and GL theories. The following concluding remarks can be drawn from the results of this study:

1. A (2-D) rotating double porous thermoelastic model with diffusion under thermal load and nonlocal can be described using a system of linear partial differential equations.
2. As expected, all field variables are continuous and satisfy the problem’s boundary conditions, indicating that the deformation of a solid depends on both the nature of the applied load and the boundary conditions.
3. All physical quantities follow similar patterns for different values of time *t*. An increase in time leads to an increase in the magnitude of all field variables.
4. The double porosity parameter due to pores and fissures (${b}_{1}$ and $\gamma$) has a significant impact on all physical quantities. It increases and decreases the profiles of normal stress, normal displacement, equilibrated stress ${\upsigma }_{y} and {\upchi }_{y}$, and diffusion, while decreasing temperature.
5. The nonlocal parameter has a clear effect on all physical quantities.
6. All field variables are highly sensitive to the rotation effect.
7. Diffusion strongly affects all considered physical fields.
8. The generalized thermoelasticity hypothesis is validated by the fact that all physical variables have nonzero values only within a limited spatial domain, as evidenced by the figures. Additionally, as the distance increases, all physical field distributions approach zero, reaching equilibrium.
9. The physical variables satisfy all the boundary conditions under investigation.
10. This research will help scientists studying thermoelasticity and using rotational motion, which is important in domains such as engineering, physics, robotics, planetary motion, subatomic particle behavior, and machine dynamics.
11. The results presented in this article will be valuable for researchers in material science, designers of new materials, and physicists, as well as those developing the theory of hyperbolic thermoelasticity. The method employed here is applicable to a wide range of problems in thermodynamics, thermoelasticity, and poroelasticity.

## Electronic supplementary material

Below is the link to the electronic supplementary material.

Supplementary Material 1

## Data Availability

The datasets used and/or analyzed during the current study available from the corresponding author on reasonable request.

## List of symbols

$$\rho$$: Material density
$$T_{^\circ }$$: Absolute temperature
$$u,v$$: Displacement components
${\Phi },{\Psi }$: Two scalar functions
$$c_{e}$$: Specific heat
$$\lambda , \mu$$: Lame’s constants
$\tau_{o} ,\tau_{1}$: Thermal relaxation times
$\tau^{^\circ }$, $\tau^{1}$: Diffusion relaxation times
$\alpha_{t}$: Thermal expansion coefficient
$\alpha_{c}$: Diffusion expansion coefficient
$q_{1}$: Volume fraction field related to pores
$q_{2}$: Volume fraction field related to fissures
K: Thermal conductivity
$${\Omega }$$: Angular velocity
$$t$$: Time
$$\sigma_{ij}$$: Stress components
$$e$$: Wave number
$$\omega$$: Angular frequency
