# Supplementary material for: Influence of nonlocal on a rotating thermoelastic medium with diffusion and double porosity
Source: Sci Rep. 2025 May 7;15:15955. doi: 10.1038/s41598-025-97334-3 (PMC12059184; doi:10.1038/s41598-025-97334-3)
Supplement: Supplementary file 1 — Supplementary Material 1 [file 41598_2025_97334_MOESM1_ESM.docx]

**Appendix I**

$a_{60}=a_{6}a_{49}+a_{43}a_{51}$,$a_{61}=a_{21}a_{42}-a_{20}a_{43}$,$a_{62}=a_{6}a_{21}$,$a_{63}=a_{43}a_{56}$,$a_{64}=a_{43}a_{57}$,

$a_{65}=a_{42}a_{49}-a_{43}a_{48}$,$a_{66}=a_{17}a_{42}-a_{16}a_{43}$,$a_{67}=a_{39}a_{49}$,$a_{68}=a_{41}a_{49}$,

$a_{69}=a_{43}a_{46}$, $a_{70}=a_{43}a_{47}$,$a_{71}=a_{42}a_{49}-a_{43}a_{48}$,$a_{72}=a_{17}a_{39}$,$a_{73}=a_{17}a_{41}$,

$a_{74}=ba_{43}-a_{34}$,$a_{75}=a_{72}+a_{43}e^{2}$,$a_{76}=a_{6}a_{49}+a_{43}a_{51}$,$a_{77}=a_{17}a_{42}-a_{16}a_{43}$,

$a_{78}=a_{6}a_{17}-a_{43}a_{54}$,$a_{79}=a_{13}a_{43}$,$a_{80}=a_{42}a_{49}-a_{43}a_{48}$,$a_{81}=a_{39}a_{49}-a_{43}a_{47}$,

$a_{82}=a_{41}a_{49}+a_{43}a_{46}$, $a_{83}=a_{21}a_{42}-a_{20}a_{43}$,$a_{84}=a_{21}a_{39}-a_{43}e^{2}$,

$a_{85}=a_{43}d-a_{21}a_{41}$,$a_{86}=a_{42}a_{49}-a_{43}a_{48}$,$a_{87}=a_{5}a_{49}+a_{43}a_{50}$,

$a_{88}=a_{17}a_{42}-a_{16}a_{43}$,$a_{89}=a_{5}a_{17}-a_{43}a_{53}$,$a_{90}=a_{43}a_{52}$,$a_{91}=a_{42}a_{49}-a_{43}a_{48}$,

$a_{92}=a_{6}a_{49}+a_{43}a_{51}$,$a_{93}=a_{21}a_{42}-a_{20}a_{43}$,$a_{94}=a_{6}a_{21}-a_{43}a_{57}$,

$a_{95}=a_{43}a_{56}$,$a_{96}=a_{42}a_{49}-a_{43}a_{48}$,$a_{97}=a_{5}a_{49}+a_{43}a_{50}$,$a_{98}=a_{21}a_{42}-a_{20}a_{43}$,

$a_{99}=a_{5}a_{21}-a_{43}a_{55}$,$a_{100}=a_{13}a_{43}$,$a_{101}=a_{42}a_{49}-a_{43}a_{48}$,$a_{102}=a_{6}a_{49}+a_{43}a_{51}$,

$a_{103}=a_{17}a_{42}-a_{16}a_{43}$,$a_{104}=a_{6}a_{17}-a_{43}a_{54}$,$a_{105}=a_{13}a_{43}$,$a_{106}=a_{42}a_{49}-a_{43}a_{48}$,

$a_{107}=a_{60}a_{61}-a_{62}+a_{63}-a_{64}$, $a_{108}=a_{65}a_{107}$,$a_{109}=a_{43}a_{107}$,

$a_{110}=a_{66}a_{67}-a_{66}a_{70}-a_{71}a_{75}$,$a_{111}=a_{66}a_{68}-a_{66}a_{79}-a_{71}a_{74}-a_{43}a_{75}$,

$a_{112}=a_{43}a_{74}$,$a_{113}=a_{70}a_{77}-a_{78}a_{80}$, $a_{114}=a_{43}a_{78}-a_{79}a_{80}$,$a_{115}=a_{43}a_{79}$,

$a_{116}=a_{81}a_{83}-a_{84}a_{86}$,$a_{117}=a_{82}a_{83}+a_{43}a_{84}-a_{85}a_{86}$,$a_{118}=a_{43}a_{85}$,

$a_{119}=a_{87}a_{88}+a_{89}a_{91}$,$a_{120}=a_{43}a_{89}-a_{90}a_{91}$,$a_{121}=a_{43}a_{90}$,$a_{122}=a_{92}a_{93}-a_{94}a_{96}$,

$a_{123}=a_{43}a_{94}-a_{95}a_{96}$,$a_{124}=a_{43}a_{95}$,$a_{125}=a_{97}a_{98}-a_{99}a_{101}$,

$a_{126}=a_{100}a_{101}+a_{43}a_{99}$,$a_{127}=a_{43}a_{100}$,$a_{128}=a_{102}a_{103}-a_{104}a_{106}$,

$a_{129}=a_{43}a_{104}+a_{105}a_{106}$,$a_{130}=a_{43}a_{105}$,$a_{131}=a_{108}a_{110}-a_{113}a_{116}$,

$a_{132}=a_{108}a_{111}-a_{109}a_{110}-a_{113}a_{117}-a_{114}a_{116}$,

$a_{133}=a_{108}a_{112}+a_{109}a_{111}+a_{113}a_{118}+a_{114}a_{117}-a_{115}a_{116}$,

$a_{134}=a_{109}a_{112}-a_{114}a_{118}+a_{115}a_{117}$, $a_{135}=a_{115}a_{118}$,$a_{136}=a_{119}a_{122}-a_{125}a_{128}$,

$a_{137}=a_{119}a_{123}+a_{120}a_{122}-a_{125}a_{129}-a_{126}a_{128}$,

$a_{138}=a_{119}a_{124}+a_{120}a_{123}-a_{121}a_{122}+a_{125}a_{130}-a_{126}a_{129}+a_{127}a_{128}$,

$a_{139}=a_{120}a_{124}-a_{121}a_{123}+a_{126}a_{130}+a_{127}a_{129}$,$a_{140}=a_{121}a_{124}+a_{127}a_{130}$,

$a_{141}=a_{39}a_{49}$,$a_{142}=a_{41}a_{49}$,$a_{143}=a_{43}a_{46}$,$a_{144}=a_{43}a_{47}$,$a_{145}=a_{21}a_{42}-a_{20}a_{43}$,

$a_{146}=a_{21}a_{39}$,$a_{147}=a_{21}a_{41}$,$a_{148}=a_{43}e^{2}$,$a_{149}=a_{43}d$,$a_{150}=a_{42}a_{43}$,$a_{151}=a_{43}a_{48}$,

$a_{152}=a_{5}a_{49}+a_{43}a_{50}$,$a_{153}=a_{21}a_{42}-a_{20}a_{43}$,$a_{154}=a_{5}a_{21}$,$a_{155}=a_{13}a_{43}$,

$a_{156}=a_{43}a_{55}$,$a_{157}=a_{42}a_{49}$,$a_{158}=a_{43}a_{48}$,$a_{159}=a_{6}a_{49}+a_{43}a_{51}$,

$a_{160}=a_{21}a_{42}-a_{20}a_{43}$,$a_{161}=a_{6}a_{21}$,$a_{162}=a_{43}a_{56}$,$a_{163}=a_{43}a_{57}$,$a_{164}=a_{42}a_{49}$,

$a_{165}=a_{43}a_{48}$,$a_{166}=a_{159}a_{160}-a_{161}-a_{163}$,$a_{167}=a_{141}a_{145}-a_{144}a_{145}$,

$a_{168}=a_{142}a_{145}+a_{143}a_{145}$,$a_{169}=a_{146}-a_{148}$,$a_{170}=a_{147}-a_{149}$,

$a_{171}=a_{152}a_{153}-a_{154}-a_{156}$, $a_{172}=a_{72}+a_{43}e^{2}$,$a_{173}=a_{73}-ba_{43}$,

$a_{174}=a_{17}a_{42}-a_{16}a_{43}$,$a_{175}=a_{5}a_{17}-a_{43}a_{53}$,$a_{176}=a_{43}a_{52}$,$a_{177}=a_{6}a_{17}-a_{43}a_{54}$,

$a_{178}=a_{13}a_{43}$,

ζ=$a_{13}^{2}a_{43}$-a_43_ a_52_ a_56_,

**B_11_** = $\frac{1}{\zeta}$ (a_6_ a_13_ a_17_+a_5_ a_13_ a_21_-a_13_ a_21_ a_35_ a_41_-a_13_ a_17_ a_36_ a_41_+d a_13_ a_35_ a_43_+b a_13_ a_36_ a_43_-$a_{13}^{2}a_{43}a_{48}$+$a_{13}^{2}a_{42}a_{49}$-a_6_ a_21_ a_52_+a_21_ a_36_ a_41_ a_52_-d a_36_ a_43_ a_52_-a_13_ a_43_ a_54_-a_13_ a_43_ a_55_-a_5_ a_17_ a_56_+a_17_ a_35_ a_41_ a_56_-b a_35_ a_43_ a_56_+a_43_ a_48_ a_52_ a_56_-a_42_ a_49_ a_52_ a_56_+a_43_ a_53_ a_56_+a_43_ a_52_ a_57_+$a_{13}^{2}a_{41}a_{49}a_{58}$

+$a_{13}^{2}a_{42}a_{42}a_{49}$-a_43_ a_46_ a_52_ a_56_ a_58_-a_41_ a_49_ a_52_ a_56_ a_58_-$a_{13}^{2}a_{41}a_{59}$+a_41_ a_52_ a_56_ a_59_),

**B_22_** =$\frac{1}{\zeta}$ (d a_6_ a_17_ a_35_-b a_6_ a_21_ a_35_-d a_5_ a_17_ a_36_+b a_5_ a_21_ a_36_+a_13_ a_21_ a_35_ a_39_+a_13_ a_17_ a_36_ a_39_+e^2^ a_13_ a_21_ a_35_ a_41_+e^2^ a_13_ a_17_ a_36_ a_41_+e^4^ $a_{13}^{2}$ a_43_-e^2^ a_13_ a_35_ a_43_-d e^2^ a_13_ a_35_ a_43_+e^2^ a_13_ a_36_ a_43_-b e^2^ a_13_ a_36_ a_43_+a_13_ a_21_ a_35_ a_42_ a_46_+a_13_ a_17_ a_36_ a_42_ a_46_-a_13_ a_20_ a_35_ a_43_ a_46_-a_13_ a_16_ a_36_ a_43_ a_46_-a_6_ a_13_ a_17_ a_48_-a_5_ a_13_ a_21_ a_48_+a_13_ a_21_ a_35_ a_41_ a_48_+a_13_ a_17_ a_36_ a_41_ a_48_-d a_13_ a_35_ a_43_ a_48_-b a_13_ a_36_ a_43_ a_48_+a_6_ a_13_ a_16_ a_49_+a_5_ a_13_ a_20_ a_49_-a_13_ a_20_ a_35_ a_41_ a_49_-a_13_ a_16_ a_36_ a_41_ a_49_+d a_13_ a_35_ a_42_ a_49_+b a_13_ a_36_ a_42_ a_49_-a_13_ a_21_ a_42_ a_50_+a_13_ a_20_ a_43_ a_50_-a_13_ a_17_ a_42_ a_51_+a_13_ a_16_ a_43_ a_51_-a_21_ a_36_ a_39_ a_52_-e^2^ a_21_ a_36_ a_41_ a_52_+e^2^ a_36_ a_43_ a_52_+d e^2^ a_36_ a_43_ a_52_-a_21_ a_36_ a_42_ a_46_ a_52_+a_20_ a_36_ a_43_ a_46_ a_52_+a_6_ a_21_ a_48_ a_52_-a_21_ a_36_ a_41_ a_48_ a_52_+d a_36_ a_43_ a_48_ a_52_-a_6_ a_20_ a_49_ a_52_+a_20_ a_36_ a_41_ a_49_ a_52_-d a_36_ a_42_ a_49_ a_52_+a_21_ a_42_ a_51_ a_52_-a_20_ a_43_ a_51_ a_52_+a_6_ a_21_ a_53_-a_21_ a_36_ a_41_ a_53_+d a_36_ a_43_ a_53_-a_5_ a_21_ a_54_+a_21_ a_35_ a_41_ a_54_-d a_35_ a_43_ a_54_+a_13_ a_43_ a_48_ a_54_-a_13_ a_42_ a_49_ a_54_-a_6_ a_17_ a_55_+a_17_ a_36_ a_41_ a_55_-b a_36_ a_43_ a_55_+a_13_ a_43_ a_48_ a_55_-a_13_ a_42_ a_49_ a_55_+a_43_ a_54_ a_55_-a_17_ a_35_ a_39_ a_56_-e^2^ a_17_ a_35_ a_41_ a_56_-e^2^ a_35_ a_43_ a_56_+b e^2^ a_35_ a_43_ a_56_-a_17_ a_35_ a_42_ a_46_ a_56_+a_16_ a_35_ a_43_ a_46_ a_56_+a_5_ a_17_ a_48_ a_56_-a_17_ a_35_ a_41_ a_48_ a_56_+b a_35_ a_43_ a_48_ a_56_-a_5_ a_16_ a_49_ a_56_+a_16_ a_35_ a_41_ a_49_ a_56_-b a_35_ a_42_ a_49_ a_56_+a_17_ a_42_ a_50_ a_56_-a_16_ a_43_ a_50_ a_56_-e^4^ a_43_ a_52_ a_56_-a_43_ a_48_ a_53_ a_56_+a_42_ a_49_ a_53_ a_56_+a_5_ a_17_ a_57_-a_17_ a_35_ a_41_ a_57_+b a_35_ a_43_ a_57_-a_43_ a_48_ a_52_ a_57_+a_42_ a_49_ a_52_ a_57_-a_43_ a_53_ a_57_+a_6_ a_13_ a_17_ a_46_ a_58_+a_5_ a_13_ a_21_ a_46_ a_58_-e^2^ $a_{13}^{2}$ a_43_ a_46_ a_58_+$a_{13}^{2}a_{43}a_{47}a_{58}$+d a_5_ a_13_ a_49_ a_58_+b a_6_ a_13_ a_49_ a_58_-$a_{13}^{2}a_{39}a_{49}a_{58}$-e^2^ $a_{13}^{2}$ a_41_ a_49_ a_58_-a_13_ a_21_ a_41_ a_50_ a_58_+d a_13_ a_43_ a_50_ a_58_-a_13_ a_17_ a_41_ a_51_ a_58_+b a_13_ a_43_ a_51_ a_58_-a_6_ a_21_ a_46_ a_52_ a_58_-d a_6_ a_49_ a_52_ a_58_+a_21_ a_41_ a_51_ a_52_ a_58_-d a_43_ a_51_ a_52_ a_58_-a_13_ a_43_ a_46_ a_54_ a_58_-a_13_ a_41_ a_49_ a_54_ a_58_-a_13_ a_43_ a_46_ a_55_ a_58_-a_13_ a_41_ a_49_ a_55_ a_58_-a_5_ a_17_ a_46_ a_56_ a_58_-b a_5_ a_49_ a_56_ a_58_+a_17_ a_41_ a_50_ a_56_ a_58_-b a_43_ a_50_ a_56_ a_58_+e^2^ a_43_ a_46_ a_52_ a_56_ a_58_-a_43_ a_47_ a_52_ a_56_ a_58_+a_39_ a_49_ a_52_ a_56_ a_58_+e^2^ a_41_ a_49_ a_52_ a_56_ a_58_+a_43_ a_46_ a_53_ a_56_ a_58_+a_41_ a_49_ a_53_ a_56_ a_58_+a_43_ a_46_ a_52_ a_57_ a_58_+a_41_ a_49_ a_52_ a_57_ a_58_-d a_5_ a_13_ a_59_-b a_6_ a_13_ a_59_+$a_{13}^{2}a_{39}a_{59}$+$a_{13}^{2}a_{42}a_{46}a_{59}$+$a_{13}^{2}a_{41}a_{48}a_{59}$+d a_6_ a_52_ a_59_+a_13_ a_41_ a_54_ a_59_+a_13_ a_41_ a_55_ a_59_+b a_5_ a_56_ a_59_-a_39_ a_52_ a_56_ a_59_-a_42_ a_46_ a_52_ a_56_ a_59_-a_41_ a_48_ a_52_ a_56_ a_59_-a_41_ a_53_ a_56_ a_59_-a_41_ a_52_ a_57_ a_59_),

**B_33_** =$\frac{1}{\zeta}$ (e^4^ a_6_ a_13_ a_17_+e^4^ a_5_ a_13_ a_21_-e^2^ a_6_ a_17_ a_35_-d e^2^ a_6_ a_17_ a_35_-e^2^ a_6_ a_21_ a_35_+b e^2^ a_6_ a_21_ a_35_+e^2^ a_5_ a_17_ a_36_+d e^2^ a_5_ a_17_ a_36_+e^2^ a_5_ a_21_ a_36_-b e^2^ a_5_ a_21_ a_36_-e^2^ a_13_ a_21_ a_35_ a_39_-e^2^ a_13_ a_17_ a_36_ a_39_+e^4^ a_13_ a_35_ a_43_-e^4^ a_13_ a_36_ a_43_-a_6_ a_17_ a_20_ a_35_ a_46_+a_6_ a_16_ a_21_ a_35_ a_46_+a_5_ a_17_ a_20_ a_36_ a_46_-a_5_ a_16_ a_21_ a_36_ a_46_-e^2^ a_13_ a_21_ a_35_ a_42_ a_46_-e^2^ a_13_ a_17_ a_36_ a_42_ a_46_+e^2^ a_13_ a_20_ a_35_ a_43_ a_46_+e^2^ a_13_ a_16_ a_36_ a_43_ a_46_+a_13_ a_21_ a_35_ a_42_ a_47_+a_13_ a_17_ a_36_ a_42_ a_47_-a_13_ a_20_ a_35_ a_43_ a_47_-a_13_ a_16_ a_36_ a_43_ a_47_-d a_6_ a_17_ a_35_ a_48_+b a_6_ a_21_ a_35_ a_48_+d a_5_ a_17_ a_36_ a_48_-b a_5_ a_21_ a_36_ a_48_-a_13_ a_21_ a_35_ a_39_ a_48_-a_13_ a_17_ a_36_ a_39_ a_48_-e^2^ a_13_ a_21_ a_35_ a_41_ a_48_-e^2^ a_13_ a_17_ a_36_ a_41_ a_48_-e^4^ $a_{13}^{2}$ a_43_ a_48_+e^2^ a_13_ a_35_ a_43_ a_48_+d e^2^ a_13_ a_35_ a_43_ a_48_-e^2^ a_13_ a_36_ a_43_ a_48_+b e^2^ a_13_ a_36_ a_43_ a_48_+d a_6_ a_16_ a_35_ a_49_-b a_6_ a_20_ a_35_ a_49_-d a_5_ a_16_ a_36_ a_49_+b a_5_ a_20_ a_36_ a_49_+a_13_ a_20_ a_35_ a_39_ a_49_+a_13_ a_16_ a_36_ a_39_ a_49_+e^2^ a_13_ a_20_ a_35_ a_41_ a_49_+e^2^ a_13_ a_16_ a_36_ a_41_ a_49_+e^4^ $a_{13}^{2}$ a_42_ a_49_-e^2^ a_13_ a_35_ a_42_ a_49_-d e^2^ a_13_ a_35_ a_42_ a_49_+e^2^ a_13_ a_36_ a_42_ a_49_-b e^2^ a_13_ a_36_ a_42_ a_49_+a_6_ a_17_ a_20_ a_50_-a_6_ a_16_ a_21_ a_50_-a_17_ a_20_ a_36_ a_41_ a_50_+a_16_ a_21_ a_36_ a_41_ a_50_+d a_17_ a_36_ a_42_ a_50_-b a_21_ a_36_ a_42_ a_50_-d a_16_ a_36_ a_43_ a_50_+b a_20_ a_36_ a_43_ a_50_-a_5_ a_17_ a_20_ a_51_+a_5_ a_16_ a_21_ a_51_+a_17_ a_20_ a_35_ a_41_ a_51_-a_16_ a_21_ a_35_ a_41_ a_51_-d a_17_ a_35_ a_42_ a_51_+b a_21_ a_35_ a_42_ a_51_+d a_16_ a_35_ a_43_ a_51_-b a_20_ a_35_ a_43_ a_51_-e^4^ a_6_ a_21_ a_52_+e^2^ a_21_ a_36_ a_39_ a_52_-e^4^ a_36_ a_43_ a_52_+e^2^ a_21_ a_36_ a_42_ a_46_ a_52_-e^2^ a_20_ a_36_ a_43_ a_46_ a_52_-a_21_ a_36_ a_42_ a_47_ a_52_+a_20_ a_36_ a_43_ a_47_ a_52_+a_21_ a_36_ a_39_ a_48_ a_52_+e^2^ a_21_ a_36_ a_41_ a_48_ a_52_-e^2^ a_36_ a_43_ a_48_ a_52_-d e^2^ a_36_ a_43_ a_48_ a_52_-a_20_ a_36_ a_39_ a_49_ a_52_-e^2^ a_20_ a_36_ a_41_ a_49_ a_52_+e^2^ a_36_ a_42_ a_49_ a_52_+d e^2^ a_36_ a_42_ a_49_ a_52_+a_21_ a_36_ a_39_ a_53_+e^2^ a_21_ a_36_ a_41_ a_53_-e^2^ a_36_ a_43_ a_53_-d e^2^ a_36_ a_43_ a_53_+a_21_ a_36_ a_42_ a_46_ a_53_-a_20_ a_36_ a_43_ a_46_ a_53_-a_6_ a_21_ a_48_ a_53_+a_21_ a_36_ a_41_ a_48_ a_53_-d a_36_ a_43_ a_48_ a_53_+a_6_ a_20_ a_49_ a_53_-a_20_ a_36_ a_41_ a_49_ a_53_+d a_36_ a_42_ a_49_ a_53_-a_21_ a_42_ a_51_ a_53_+a_20_ a_43_ a_51_ a_53_-a_21_ a_35_ a_39_ a_54_-e^2^ a_21_ a_35_ a_41_ a_54_-e^4^ a_13_ a_43_ a_54_+e^2^ a_35_ a_43_ a_54_+d e^2^ a_35_ a_43_ a_54_-a_21_ a_35_ a_42_ a_46_ a_54_+a_20_ a_35_ a_43_ a_46_ a_54_+a_5_ a_21_ a_48_ a_54_-a_21_ a_35_ a_41_ a_48_ a_54_+d a_35_ a_43_ a_48_ a_54_-a_5_ a_20_ a_49_ a_54_+a_20_ a_35_ a_41_ a_49_ a_54_-d a_35_ a_42_ a_49_ a_54_+a_21_ a_42_ a_50_ a_54_-a_20_ a_43_ a_50_ a_54_-a_17_ a_36_ a_39_ a_55_-e^2^ a_17_ a_36_ a_41_ a_55_-e^4^ a_13_ a_43_ a_55_-e^2^ a_36_ a_43_ a_55_+b e^2^ a_36_ a_43_ a_55_-a_17_ a_36_ a_42_ a_46_ a_55_+a_16_ a_36_ a_43_ a_46_ a_55_+a_6_ a_17_ a_48_ a_55_-a_17_ a_36_ a_41_ a_48_ a_55_+b a_36_ a_43_ a_48_ a_55_-a_6_ a_16_ a_49_ a_55_+a_16_ a_36_ a_41_ a_49_ a_55_-b a_36_ a_42_ a_49_ a_55_+a_17_ a_42_ a_51_ a_55_-a_16_ a_43_ a_51_ a_55_-a_43_ a_48_ a_54_ a_55_+a_42_ a_49_ a_54_ a_55_-e^4^ a_5_ a_17_ a_56_+e^2^ a_17_ a_35_ a_39_ a_56_+e^4^ a_35_ a_43_ a_56_+e^2^ a_17_ a_35_ a_42_ a_46_ a_56_-e^2^ a_16_ a_35_ a_43_ a_46_ a_56_-a_17_ a_35_ a_42_ a_47_ a_56_+a_16_ a_35_ a_43_ a_47_ a_56_+a_17_ a_35_ a_39_ a_48_ a_56_+e^2^ a_17_ a_35_ a_41_ a_48_ a_56_+e^2^ a_35_ a_43_ a_48_ a_56_-b e^2^ a_35_ a_43_ a_48_ a_56_-a_16_ a_35_ a_39_ a_49_ a_56_-e^2^ a_16_ a_35_ a_41_ a_49_ a_56_-e^2^ a_35_ a_42_ a_49_ a_56_+b e^2^ a_35_ a_42_ a_49_ a_56_+e^4^ a_43_ a_48_ a_52_ a_56_-e^4^ a_42_ a_49_ a_52_ a_56_+e^4^ a_43_ a_53_ a_56_+a_17_ a_35_ a_39_ a_57_+e^2^ a_17_ a_35_ a_41_ a_57_+e^2^ a_35_ a_43_ a_57_-b e^2^ a_35_ a_43_ a_57_+a_17_ a_35_ a_42_ a_46_ a_57_-a_16_ a_35_ a_43_ a_46_ a_57_-a_5_ a_17_ a_48_ a_57_+a_17_ a_35_ a_41_ a_48_ a_57_-b a_35_ a_43_ a_48_ a_57_+a_5_ a_16_ a_49_ a_57_-a_16_ a_35_ a_41_ a_49_ a_57_+b a_35_ a_42_ a_49_ a_57_-a_17_ a_42_ a_50_ a_57_+a_16_ a_43_ a_50_ a_57_+e^4^ a_43_ a_52_ a_57_+a_43_ a_48_ a_53_ a_57_-a_42_ a_49_ a_53_ a_57_-e^2^ a_6_ a_13_ a_17_ a_46_ a_58_-e^2^ a_5_ a_13_ a_21_ a_46_ a_58_+a_6_ a_13_ a_17_ a_47_ a_58_+a_5_ a_13_ a_21_ a_47_ a_58_-e^2^ $a_{13}^{2}$ a_43_ a_47_ a_58_-e^2^ a_5_ a_13_ a_49_ a_58_-d e^2^ a_5_ a_13_ a_49_ a_58_+e^2^ a_6_ a_13_ a_49_ a_58_-b e^2^ a_6_ a_13_ a_49_ a_58_+e^2^ $a_{13}^{2}$ a_39_ a_49_ a_58_+d a_6_ a_17_ a_50_ a_58_-b a_6_ a_21_ a_50_ a_58_+a_13_ a_21_ a_39_ a_50_ a_58_+e^2^ a_13_ a_21_ a_41_ a_50_ a_58_-e^2^ a_13_ a_43_ a_50_ a_58_-d e^2^ a_13_ a_43_ a_50_ a_58_-d a_5_ a_17_ a_51_ a_58_+b a_5_ a_21_ a_51_ a_58_+a_13_ a_17_ a_39_ a_51_ a_58_+e^2^ a_13_ a_17_ a_41_ a_51_ a_58_+e^2^ a_13_ a_43_ a_51_ a_58_-b e^2^ a_13_ a_43_ a_51_ a_58_+e^2^ a_6_ a_21_ a_46_ a_52_ a_58_-a_6_ a_21_ a_47_ a_52_ a_58_+e^2^ a_6_ a_49_ a_52_ a_58_+d e^2^ a_6_ a_49_ a_52_ a_58_-a_21_ a_39_ a_51_ a_52_ a_58_-e^2^ a_21_ a_41_ a_51_ a_52_ a_58_+e^2^ a_43_ a_51_ a_52_ a_58_+d e^2^ a_43_ a_51_ a_52_ a_58_+a_6_ a_21_ a_46_ a_53_ a_58_+d a_6_ a_49_ a_53_ a_58_-a_21_ a_41_ a_51_ a_53_ a_58_+d a_43_ a_51_ a_53_ a_58_-a_5_ a_21_ a_46_ a_54_ a_58_+e^2^ a_13_ a_43_ a_46_ a_54_ a_58_-a_13_ a_43_ a_47_ a_54_ a_58_-d a_5_ a_49_ a_54_ a_58_+a_13_ a_39_ a_49_ a_54_ a_58_+e^2^ a_13_ a_41_ a_49_ a_54_ a_58_+a_21_ a_41_ a_50_ a_54_ a_58_-d a_43_ a_50_ a_54_ a_58_-a_6_ a_17_ a_46_ a_55_ a_58_+e^2^ a_13_ a_43_ a_46_ a_55_ a_58_-a_13_ a_43_ a_47_ a_55_ a_58_-b a_6_ a_49_ a_55_ a_58_+a_13_ a_39_ a_49_ a_55_ a_58_+e^2^ a_13_ a_41_ a_49_ a_55_ a_58_+a_17_ a_41_ a_51_ a_55_ a_58_-b a_43_ a_51_ a_55_ a_58_+a_43_ a_46_ a_54_ a_55_ a_58_+a_41_ a_49_ a_54_ a_55_ a_58_+e^2^ a_5_ a_17_ a_46_ a_56_ a_58_-a_5_ a_17_ a_47_ a_56_ a_58_-e^2^ a_5_ a_49_ a_56_ a_58_+b e^2^ a_5_ a_49_ a_56_ a_58_-a_17_ a_39_ a_50_ a_56_ a_58_-e^2^ a_17_ a_41_ a_50_ a_56_ a_58_-e^2^ a_43_ a_50_ a_56_ a_58_+b e^2^ a_43_ a_50_ a_56_ a_58_+e^2^ a_43_ a_47_ a_52_ a_56_ a_58_-e^2^ a_39_ a_49_ a_52_ a_56_ a_58_-e^2^ a_43_ a_46_ a_53_ a_56_ a_58_+a_43_ a_47_ a_53_ a_56_ a_58_-a_39_ a_49_ a_53_ a_56_ a_58_-e^2^ a_41_ a_49_ a_53_ a_56_ a_58_+a_5_ a_17_ a_46_ a_57_ a_58_+b a_5_ a_49_ a_57_ a_58_-a_17_ a_41_ a_50_ a_57_ a_58_+b a_43_ a_50_ a_57_ a_58_-e^2^ a_43_ a_46_ a_52_ a_57_ a_58_+a_43_ a_47_ a_52_ a_57_ a_58_-a_39_ a_49_ a_52_ a_57_ a_58_-e^2^ a_41_ a_49_ a_52_ a_57_ a_58_-a_43_ a_46_ a_53_ a_57_ a_58_-a_41_ a_49_ a_53_ a_57_ a_58_+e^2^ a_5_ a_13_ a_59_-e^2^ a_6_ a_13_ a_59_+a_6_ a_13_ a_16_ a_46_ a_59_+a_5_ a_13_ a_20_ a_46_ a_59_+$a_{13}^{2}a_{42}a_{47}a_{59}$+d a_5_ a_13_ a_48_ a_59_+b a_6_ a_13_ a_48_ a_59_-$a_{13}^{2}a_{39}a_{48}a_{59}$-a_13_ a_20_ a_41_ a_50_ a_59_+d a_13_ a_42_ a_50_ a_59_-a_13_ a_16_ a_41_ a_51_ a_59_+b a_13_ a_42_ a_51_ a_59_-e^2^ a_6_ a_52_ a_59_-a_6_ a_20_ a_46_ a_52_ a_59_-d a_6_ a_48_ a_52_ a_59_+a_20_ a_41_ a_51_ a_52_ a_59_-d a_42_ a_51_ a_52_ a_59_-d a_6_ a_53_ a_59_+d a_5_ a_54_ a_59_-a_13_ a_39_ a_54_ a_59_-a_13_ a_42_ a_46_ a_54_ a_59_-a_13_ a_41_ a_48_ a_54_ a_59_+b a_6_ a_55_ a_59_-a_13_ a_39_ a_55_ a_59_-a_13_ a_42_ a_46_ a_55_ a_59_-a_13_ a_41_ a_48_ a_55_ a_59_-a_41_ a_54_ a_55_ a_59_+e^2^ a_5_ a_56_ a_59_-a_5_ a_16_ a_46_ a_56_ a_59_-b a_5_ a_48_ a_56_ a_59_+a_16_ a_41_ a_50_ a_56_ a_59_-b a_42_ a_50_ a_56_ a_59_-a_42_ a_47_ a_52_ a_56_ a_59_+a_39_ a_48_ a_52_ a_56_ a_59_+a_39_ a_53_ a_56_ a_59_+a_42_ a_46_ a_53_ a_56_ a_59_+a_41_ a_48_ a_53_ a_56_ a_59_-b a_5_ a_57_ a_59_+a_39_ a_52_ a_57_ a_59_+a_42_ a_46_ a_52_ a_57_ a_59_+a_41_ a_48_ a_52_ a_57_ a_59_+a_41_ a_53_ a_57_ a_59_),

**B_44_** = $\frac{1}{\zeta}$ (e^4^ a_6_ a_17_ a_35_+e^4^ a_6_ a_21_ a_35_-e^4^ a_5_ a_17_ a_36_-e^4^ a_5_ a_21_ a_36_+e^2^ a_6_ a_17_ a_20_ a_35_ a_46_-e^2^ a_6_ a_16_ a_21_ a_35_ a_46_-e^2^ a_5_ a_17_ a_20_ a_36_ a_46_+e^2^ a_5_ a_16_ a_21_ a_36_ a_46_-a_6_ a_17_ a_20_ a_35_ a_47_+a_6_ a_16_ a_21_ a_35_ a_47_+a_5_ a_17_ a_20_ a_36_ a_47_-a_5_ a_16_ a_21_ a_36_ a_47_-e^2^ a_13_ a_21_ a_35_ a_42_ a_47_-e^2^ a_13_ a_17_ a_36_ a_42_ a_47_+e^2^ a_13_ a_20_ a_35_ a_43_ a_47_+e^2^ a_13_ a_16_ a_36_ a_43_ a_47_-e^4^ a_6_ a_13_ a_17_ a_48_-e^4^ a_5_ a_13_ a_21_ a_48_+e^2^ a_6_ a_17_ a_35_ a_48_+d e^2^ a_6_ a_17_ a_35_ a_48_+e^2^ a_6_ a_21_ a_35_ a_48_-b e^2^ a_6_ a_21_ a_35_ a_48_-e^2^ a_5_ a_17_ a_36_ a_48_-d e^2^ a_5_ a_17_ a_36_ a_48_-e^2^ a_5_ a_21_ a_36_ a_48_+b e^2^ a_5_ a_21_ a_36_ a_48_+e^2^ a_13_ a_21_ a_35_ a_39_ a_48_+e^2^ a_13_ a_17_ a_36_ a_39_ a_48_-e^4^ a_13_ a_35_ a_43_ a_48_+e^4^ a_13_ a_36_ a_43_ a_48_+e^4^ a_6_ a_13_ a_16_ a_49_+e^4^ a_5_ a_13_ a_20_ a_49_-e^2^ a_6_ a_16_ a_35_ a_49_-d e^2^ a_6_ a_16_ a_35_ a_49_-e^2^ a_6_ a_20_ a_35_ a_49_+b e^2^ a_6_ a_20_ a_35_ a_49_+e^2^ a_5_ a_16_ a_36_ a_49_+d e^2^ a_5_ a_16_ a_36_ a_49_+e^2^ a_5_ a_20_ a_36_ a_49_-b e^2^ a_5_ a_20_ a_36_ a_49_-e^2^ a_13_ a_20_ a_35_ a_39_ a_49_-e^2^ a_13_ a_16_ a_36_ a_39_ a_49_+e^4^ a_13_ a_35_ a_42_ a_49_-e^4^ a_13_ a_36_ a_42_ a_49_+a_17_ a_20_ a_36_ a_39_ a_50_-a_16_ a_21_ a_36_ a_39_ a_50_+e^2^ a_17_ a_20_ a_36_ a_41_ a_50_-e^2^ a_16_ a_21_ a_36_ a_41_ a_50_-e^4^ a_13_ a_21_ a_42_ a_50_-e^2^ a_17_ a_36_ a_42_ a_50_-d e^2^ a_17_ a_36_ a_42_ a_50_-e^2^ a_21_ a_36_ a_42_ a_50_+b e^2^ a_21_ a_36_ a_42_ a_50_+e^4^ a_13_ a_20_ a_43_ a_50_+e^2^ a_16_ a_36_ a_43_ a_50_+d e^2^ a_16_ a_36_ a_43_ a_50_+e^2^ a_20_ a_36_ a_43_ a_50_-b e^2^ a_20_ a_36_ a_43_ a_50_-a_17_ a_20_ a_35_ a_39_ a_51_+a_16_ a_21_ a_35_ a_39_ a_51_-e^2^ a_17_ a_20_ a_35_ a_41_ a_51_+e^2^ a_16_ a_21_ a_35_ a_41_ a_51_-e^4^ a_13_ a_17_ a_42_ a_51_+e^2^ a_17_ a_35_ a_42_ a_51_+d e^2^ a_17_ a_35_ a_42_ a_51_+e^2^ a_21_ a_35_ a_42_ a_51_-b e^2^ a_21_ a_35_ a_42_ a_51_+e^4^ a_13_ a_16_ a_43_ a_51_-e^2^ a_16_ a_35_ a_43_ a_51_-d e^2^ a_16_ a_35_ a_43_ a_51_-e^2^ a_20_ a_35_ a_43_ a_51_+b e^2^ a_20_ a_35_ a_43_ a_51_+e^2^ a_21_ a_36_ a_42_ a_47_ a_52_-e^2^ a_20_ a_36_ a_43_ a_47_ a_52_+e^4^ a_6_ a_21_ a_48_ a_52_-e^2^ a_21_ a_36_ a_39_ a_48_ a_52_+e^4^ a_36_ a_43_ a_48_ a_52_-e^4^ a_6_ a_20_ a_49_ a_52_+e^2^ a_20_ a_36_ a_39_ a_49_ a_52_-e^4^ a_36_ a_42_ a_49_ a_52_+e^4^ a_21_ a_42_ a_51_ a_52_-e^4^ a_20_ a_43_ a_51_ a_52_+e^4^ a_6_ a_21_ a_53_-e^2^ a_21_ a_36_ a_39_ a_53_+e^4^ a_36_ a_43_ a_53_-e^2^ a_21_ a_36_ a_42_ a_46_ a_53_+e^2^ a_20_ a_36_ a_43_ a_46_ a_53_+a_21_ a_36_ a_42_ a_47_ a_53_-a_20_ a_36_ a_43_ a_47_ a_53_-a_21_ a_36_ a_39_ a_48_ a_53_-e^2^ a_21_ a_36_ a_41_ a_48_ a_53_+e^2^ a_36_ a_43_ a_48_ a_53_+d e^2^ a_36_ a_43_ a_48_ a_53_+a_20_ a_36_ a_39_ a_49_ a_53_+e^2^ a_20_ a_36_ a_41_ a_49_ a_53_-e^2^ a_36_ a_42_ a_49_ a_53_-d e^2^ a_36_ a_42_ a_49_ a_53_-e^4^ a_5_ a_21_ a_54_+e^2^ a_21_ a_35_ a_39_ a_54_-e^4^ a_35_ a_43_ a_54_+e^2^ a_21_ a_35_ a_42_ a_46_ a_54_-e^2^ a_20_ a_35_ a_43_ a_46_ a_54_-a_21_ a_35_ a_42_ a_47_ a_54_+a_20_ a_35_ a_43_ a_47_ a_54_+a_21_ a_35_ a_39_ a_48_ a_54_+e^2^ a_21_ a_35_ a_41_ a_48_ a_54_+e^4^ a_13_ a_43_ a_48_ a_54_-e^2^ a_35_ a_43_ a_48_ a_54_-d e^2^ a_35_ a_43_ a_48_ a_54_-a_20_ a_35_ a_39_ a_49_ a_54_-e^2^ a_20_ a_35_ a_41_ a_49_ a_54_-e^4^ a_13_ a_42_ a_49_ a_54_+e^2^ a_35_ a_42_ a_49_ a_54_+d e^2^ a_35_ a_42_ a_49_ a_54_-e^4^ a_6_ a_17_ a_55_+e^2^ a_17_ a_36_ a_39_ a_55_+e^4^ a_36_ a_43_ a_55_+e^2^ a_17_ a_36_ a_42_ a_46_ a_55_-e^2^ a_16_ a_36_ a_43_ a_46_ a_55_-a_17_ a_36_ a_42_ a_47_ a_55_+a_16_ a_36_ a_43_ a_47_ a_55_+a_17_ a_36_ a_39_ a_48_ a_55_+e^2^ a_17_ a_36_ a_41_ a_48_ a_55_+e^4^ a_13_ a_43_ a_48_ a_55_+e^2^ a_36_ a_43_ a_48_ a_55_-b e^2^ a_36_ a_43_ a_48_ a_55_-a_16_ a_36_ a_39_ a_49_ a_55_-e^2^ a_16_ a_36_ a_41_ a_49_ a_55_-e^4^ a_13_ a_42_ a_49_ a_55_-e^2^ a_36_ a_42_ a_49_ a_55_+b e^2^ a_36_ a_42_ a_49_ a_55_+e^4^ a_43_ a_54_ a_55_+e^2^ a_17_ a_35_ a_42_ a_47_ a_56_-e^2^ a_16_ a_35_ a_43_ a_47_ a_56_+e^4^ a_5_ a_17_ a_48_ a_56_-e^2^ a_17_ a_35_ a_39_ a_48_ a_56_-e^4^ a_35_ a_43_ a_48_ a_56_-e^4^ a_5_ a_16_ a_49_ a_56_+e^2^ a_16_ a_35_ a_39_ a_49_ a_56_+e^4^ a_35_ a_42_ a_49_ a_56_+e^4^ a_17_ a_42_ a_50_ a_56_-e^4^ a_16_ a_43_ a_50_ a_56_-e^4^ a_43_ a_48_ a_53_ a_56_+e^4^ a_42_ a_49_ a_53_ a_56_+e^4^ a_5_ a_17_ a_57_-e^2^ a_17_ a_35_ a_39_ a_57_-e^4^ a_35_ a_43_ a_57_-e^2^ a_17_ a_35_ a_42_ a_46_ a_57_+e^2^ a_16_ a_35_ a_43_ a_46_ a_57_+a_17_ a_35_ a_42_ a_47_ a_57_-a_16_ a_35_ a_43_ a_47_ a_57_-a_17_ a_35_ a_39_ a_48_ a_57_-e^2^ a_17_ a_35_ a_41_ a_48_ a_57_-e^2^ a_35_ a_43_ a_48_ a_57_+b e^2^ a_35_ a_43_ a_48_ a_57_+a_16_ a_35_ a_39_ a_49_ a_57_+e^2^ a_16_ a_35_ a_41_ a_49_ a_57_+e^2^ a_35_ a_42_ a_49_ a_57_-b e^2^ a_35_ a_42_ a_49_ a_57_-e^4^ a_43_ a_48_ a_52_ a_57_+e^4^ a_42_ a_49_ a_52_ a_57_-e^4^ a_43_ a_53_ a_57_-e^2^ a_6_ a_13_ a_17_ a_47_ a_58_-e^2^ a_5_ a_13_ a_21_ a_47_ a_58_+e^4^ a_5_ a_13_ a_49_ a_58_-e^4^ a_6_ a_13_ a_49_ a_58_-e^2^ a_6_ a_17_ a_50_ a_58_-d e^2^ a_6_ a_17_ a_50_ a_58_-e^2^ a_6_ a_21_ a_50_ a_58_+b e^2^ a_6_ a_21_ a_50_ a_58_-e^2^ a_13_ a_21_ a_39_ a_50_ a_58_+e^4^ a_13_ a_43_ a_50_ a_58_+e^2^ a_5_ a_17_ a_51_ a_58_+d e^2^ a_5_ a_17_ a_51_ a_58_+e^2^ a_5_ a_21_ a_51_ a_58_-b e^2^ a_5_ a_21_ a_51_ a_58_-e^2^ a_13_ a_17_ a_39_ a_51_ a_58_-e^4^ a_13_ a_43_ a_51_ a_58_+e^2^ a_6_ a_21_ a_47_ a_52_ a_58_-e^4^ a_6_ a_49_ a_52_ a_58_+e^2^ a_21_ a_39_ a_51_ a_52_ a_58_-e^4^ a_43_ a_51_ a_52_ a_58_-e^2^ a_6_ a_21_ a_46_ a_53_ a_58_+a_6_ a_21_ a_47_ a_53_ a_58_-e^2^ a_6_ a_49_ a_53_ a_58_-d e^2^ a_6_ a_49_ a_53_ a_58_+a_21_ a_39_ a_51_ a_53_ a_58_+e^2^ a_21_ a_41_ a_51_ a_53_ a_58_-e^2^ a_43_ a_51_ a_53_ a_58_-d e^2^ a_43_ a_51_ a_53_ a_58_+e^2^ a_5_ a_21_ a_46_ a_54_ a_58_-a_5_ a_21_ a_47_ a_54_ a_58_+e^2^ a_13_ a_43_ a_47_ a_54_ a_58_+e^2^ a_5_ a_49_ a_54_ a_58_+d e^2^ a_5_ a_49_ a_54_ a_58_-e^2^ a_13_ a_39_ a_49_ a_54_ a_58_-a_21_ a_39_ a_50_ a_54_ a_58_-e^2^ a_21_ a_41_ a_50_ a_54_ a_58_+e^2^ a_43_ a_50_ a_54_ a_58_+d e^2^ a_43_ a_50_ a_54_ a_58_+e^2^ a_6_ a_17_ a_46_ a_55_ a_58_-a_6_ a_17_ a_47_ a_55_ a_58_+e^2^ a_13_ a_43_ a_47_ a_55_ a_58_-e^2^ a_6_ a_49_ a_55_ a_58_+b e^2^ a_6_ a_49_ a_55_ a_58_-e^2^ a_13_ a_39_ a_49_ a_55_ a_58_-a_17_ a_39_ a_51_ a_55_ a_58_-e^2^ a_17_ a_41_ a_51_ a_55_ a_58_-e^2^ a_43_ a_51_ a_55_ a_58_+b e^2^ a_43_ a_51_ a_55_ a_58_-e^2^ a_43_ a_46_ a_54_ a_55_ a_58_+a_43_ a_47_ a_54_ a_55_ a_58_-a_39_ a_49_ a_54_ a_55_ a_58_-e^2^ a_41_ a_49_ a_54_ a_55_ a_58_+e^2^ a_5_ a_17_ a_47_ a_56_ a_58_+e^4^ a_5_ a_49_ a_56_ a_58_+e^2^ a_17_ a_39_ a_50_ a_56_ a_58_+e^4^ a_43_ a_50_ a_56_ a_58_-e^2^ a_43_ a_47_ a_53_ a_56_ a_58_+e^2^ a_39_ a_49_ a_53_ a_56_ a_58_-e^2^ a_5_ a_17_ a_46_ a_57_ a_58_+a_5_ a_17_ a_47_ a_57_ a_58_+e^2^ a_5_ a_49_ a_57_ a_58_-b e^2^ a_5_ a_49_ a_57_ a_58_+a_17_ a_39_ a_50_ a_57_ a_58_+e^2^ a_17_ a_41_ a_50_ a_57_ a_58_+e^2^ a_43_ a_50_ a_57_ a_58_-b e^2^ a_43_ a_50_ a_57_ a_58_-e^2^ a_43_ a_47_ a_52_ a_57_ a_58_+e^2^ a_39_ a_49_ a_52_ a_57_ a_58_+e^2^ a_43_ a_46_ a_53_ a_57_ a_58_-a_43_ a_47_ a_53_ a_57_ a_58_+a_39_ a_49_ a_53_ a_57_ a_58_+e^2^ a_41_ a_49_ a_53_ a_57_ a_58_+a_6_ a_13_ a_16_ a_47_ a_59_+a_5_ a_13_ a_20_ a_47_ a_59_-e^2^ a_5_ a_13_ a_48_ a_59_+e^2^ a_6_ a_13_ a_48_ a_59_+d a_6_ a_16_ a_50_ a_59_-b a_6_ a_20_ a_50_ a_59_+a_13_ a_20_ a_39_ a_50_ a_59_-e^2^ a_13_ a_42_ a_50_ a_59_-d a_5_ a_16_ a_51_ a_59_+b a_5_ a_20_ a_51_ a_59_+a_13_ a_16_ a_39_ a_51_ a_59_+e^2^ a_13_ a_42_ a_51_ a_59_-a_6_ a_20_ a_47_ a_52_ a_59_+e^2^ a_6_ a_48_ a_52_ a_59_-a_20_ a_39_ a_51_ a_52_ a_59_+e^2^ a_42_ a_51_ a_52_ a_59_+e^2^ a_6_ a_53_ a_59_+a_6_ a_20_ a_46_ a_53_ a_59_+d a_6_ a_48_ a_53_ a_59_-a_20_ a_41_ a_51_ a_53_ a_59_+d a_42_ a_51_ a_53_ a_59_-e^2^ a_5_ a_54_ a_59_-a_5_ a_20_ a_46_ a_54_ a_59_-a_13_ a_42_ a_47_ a_54_ a_59_-d a_5_ a_48_ a_54_ a_59_+a_13_ a_39_ a_48_ a_54_ a_59_+a_20_ a_41_ a_50_ a_54_ a_59_-d a_42_ a_50_ a_54_ a_59_+e^2^ a_6_ a_55_ a_59_-a_6_ a_16_ a_46_ a_55_ a_59_-a_13_ a_42_ a_47_ a_55_ a_59_-b a_6_ a_48_ a_55_ a_59_+a_13_ a_39_ a_48_ a_55_ a_59_+a_16_ a_41_ a_51_ a_55_ a_59_-b a_42_ a_51_ a_55_ a_59_+a_39_ a_54_ a_55_ a_59_+a_42_ a_46_ a_54_ a_55_ a_59_+a_41_ a_48_ a_54_ a_55_ a_59_-a_5_ a_16_ a_47_ a_56_ a_59_-e^2^ a_5_ a_48_ a_56_ a_59_-a_16_ a_39_ a_50_ a_56_ a_59_-e^2^ a_42_ a_50_ a_56_ a_59_+a_42_ a_47_ a_53_ a_56_ a_59_-a_39_ a_48_ a_53_ a_56_ a_59_-e^2^ a_5_ a_57_ a_59_+a_5_ a_16_ a_46_ a_57_ a_59_+b a_5_ a_48_ a_57_ a_59_-a_16_ a_41_ a_50_ a_57_ a_59_+b a_42_ a_50_ a_57_ a_59_+a_42_ a_47_ a_52_ a_57_ a_59_-a_39_ a_48_ a_52_ a_57_ a_59_-a_39_ a_53_ a_57_ a_59_-a_42_ a_46_ a_53_ a_57_ a_59_-a_41_ a_48_ a_53_ a_57_ a_59_),

**B_55_**  =$\frac{1}{\zeta}$ (e^2^ a_6_ a_17_ a_20_ a_35_ a_47_-e^2^ a_6_ a_16_ a_21_ a_35_ a_47_-e^2^ a_5_ a_17_ a_20_ a_36_ a_47_+e^2^ a_5_ a_16_ a_21_ a_36_ a_47_-e^4^ a_6_ a_17_ a_35_ a_48_-e^4^ a_6_ a_21_ a_35_ a_48_+e^4^ a_5_ a_17_ a_36_ a_48_+e^4^ a_5_ a_21_ a_36_ a_48_+e^4^ a_6_ a_16_ a_35_ a_49_+e^4^ a_6_ a_20_ a_35_ a_49_-e^4^ a_5_ a_16_ a_36_ a_49_-e^4^ a_5_ a_20_ a_36_ a_49_+e^4^ a_6_ a_17_ a_20_ a_50_-e^4^ a_6_ a_16_ a_21_ a_50_-e^2^ a_17_ a_20_ a_36_ a_39_ a_50_+e^2^ a_16_ a_21_ a_36_ a_39_ a_50_+e^4^ a_17_ a_36_ a_42_ a_50_+e^4^ a_21_ a_36_ a_42_ a_50_-e^4^ a_16_ a_36_ a_43_ a_50_-e^4^ a_20_ a_36_ a_43_ a_50_-e^4^ a_5_ a_17_ a_20_ a_51_+e^4^ a_5_ a_16_ a_21_ a_51_+e^2^ a_17_ a_20_ a_35_ a_39_ a_51_-e^2^ a_16_ a_21_ a_35_ a_39_ a_51_-e^4^ a_17_ a_35_ a_42_ a_51_-e^4^ a_21_ a_35_ a_42_ a_51_+e^4^ a_16_ a_35_ a_43_ a_51_+e^4^ a_20_ a_35_ a_43_ a_51_-e^2^ a_21_ a_36_ a_42_ a_47_ a_53_+e^2^ a_20_ a_36_ a_43_ a_47_ a_53_-e^4^ a_6_ a_21_ a_48_ a_53_+e^2^ a_21_ a_36_ a_39_ a_48_ a_53_-e^4^ a_36_ a_43_ a_48_ a_53_+e^4^ a_6_ a_20_ a_49_ a_53_-e^2^ a_20_ a_36_ a_39_ a_49_ a_53_+e^4^ a_36_ a_42_ a_49_ a_53_-e^4^ a_21_ a_42_ a_51_ a_53_+e^4^ a_20_ a_43_ a_51_ a_53_+e^2^ a_21_ a_35_ a_42_ a_47_ a_54_-e^2^ a_20_ a_35_ a_43_ a_47_ a_54_+e^4^ a_5_ a_21_ a_48_ a_54_-e^2^ a_21_ a_35_ a_39_ a_48_ a_54_+e^4^ a_35_ a_43_ a_48_ a_54_-e^4^ a_5_ a_20_ a_49_ a_54_+e^2^ a_20_ a_35_ a_39_ a_49_ a_54_-e^4^ a_35_ a_42_ a_49_ a_54_+e^4^ a_21_ a_42_ a_50_ a_54_-e^4^ a_20_ a_43_ a_50_ a_54_+e^2^ a_17_ a_36_ a_42_ a_47_ a_55_-e^2^ a_16_ a_36_ a_43_ a_47_ a_55_+e^4^ a_6_ a_17_ a_48_ a_55_-e^2^ a_17_ a_36_ a_39_ a_48_ a_55_-e^4^ a_36_ a_43_ a_48_ a_55_-e^4^ a_6_ a_16_ a_49_ a_55_+e^2^ a_16_ a_36_ a_39_ a_49_ a_55_+e^4^ a_36_ a_42_ a_49_ a_55_+e^4^ a_17_ a_42_ a_51_ a_55_-e^4^ a_16_ a_43_ a_51_ a_55_-e^4^ a_43_ a_48_ a_54_ a_55_+e^4^ a_42_ a_49_ a_54_ a_55_-e^2^ a_17_ a_35_ a_42_ a_47_ a_57_+e^2^ a_16_ a_35_ a_43_ a_47_ a_57_-e^4^ a_5_ a_17_ a_48_ a_57_+e^2^ a_17_ a_35_ a_39_ a_48_ a_57_+e^4^ a_35_ a_43_ a_48_ a_57_+e^4^ a_5_ a_16_ a_49_ a_57_-e^2^ a_16_ a_35_ a_39_ a_49_ a_57_-e^4^ a_35_ a_42_ a_49_ a_57_-e^4^ a_17_ a_42_ a_50_ a_57_+e^4^ a_16_ a_43_ a_50_ a_57_+e^4^ a_43_ a_48_ a_53_ a_57_-e^4^ a_42_ a_49_ a_53_ a_57_+e^4^ a_6_ a_17_ a_50_ a_58_+e^4^ a_6_ a_21_ a_50_ a_58_-e^4^ a_5_ a_17_ a_51_ a_58_-e^4^ a_5_ a_21_ a_51_ a_58_-e^2^ a_6_ a_21_ a_47_ a_53_ a_58_+e^4^ a_6_ a_49_ a_53_ a_58_-e^2^ a_21_ a_39_ a_51_ a_53_ a_58_+e^4^ a_43_ a_51_ a_53_ a_58_+e^2^ a_5_ a_21_ a_47_ a_54_ a_58_-e^4^ a_5_ a_49_ a_54_ a_58_+e^2^ a_21_ a_39_ a_50_ a_54_ a_58_-e^4^ a_43_ a_50_ a_54_ a_58_+e^2^ a_6_ a_17_ a_47_ a_55_ a_58_+e^4^ a_6_ a_49_ a_55_ a_58_+e^2^ a_17_ a_39_ a_51_ a_55_ a_58_+e^4^ a_43_ a_51_ a_55_ a_58_-e^2^ a_43_ a_47_ a_54_ a_55_ a_58_+e^2^ a_39_ a_49_ a_54_ a_55_ a_58_-e^2^ a_5_ a_17_ a_47_ a_57_ a_58_-e^4^ a_5_ a_49_ a_57_ a_58_-e^2^ a_17_ a_39_ a_50_ a_57_ a_58_-e^4^ a_43_ a_50_ a_57_ a_58_+e^2^ a_43_ a_47_ a_53_ a_57_ a_58_-e^2^ a_39_ a_49_ a_53_ a_57_ a_58_-e^2^ a_6_ a_16_ a_50_ a_59_-e^2^ a_6_ a_20_ a_50_ a_59_+e^2^ a_5_ a_16_ a_51_ a_59_+e^2^ a_5_ a_20_ a_51_ a_59_+a_6_ a_20_ a_47_ a_53_ a_59_-e^2^ a_6_ a_48_ a_53_ a_59_+a_20_ a_39_ a_51_ a_53_ a_59_-e^2^ a_42_ a_51_ a_53_ a_59_-a_5_ a_20_ a_47_ a_54_ a_59_+e^2^ a_5_ a_48_ a_54_ a_59_-a_20_ a_39_ a_50_ a_54_ a_59_+e^2^ a_42_ a_50_ a_54_ a_59_-a_6_ a_16_ a_47_ a_55_ a_59_-e^2^ a_6_ a_48_ a_55_ a_59_-a_16_ a_39_ a_51_ a_55_ a_59_-e^2^ a_42_ a_51_ a_55_ a_59_+a_42_ a_47_ a_54_ a_55_ a_59_-a_39_ a_48_ a_54_ a_55_ a_59_+a_5_ a_16_ a_47_ a_57_ a_59_+e^2^ a_5_ a_48_ a_57_ a_59_+a_16_ a_39_ a_50_ a_57_ a_59_+e^2^ a_42_ a_50_ a_57_ a_59_-a_42_ a_47_ a_53_ a_57_ a_59_+a_39_ a_48_ a_53_ a_57_ a_59_).

**Appendix II**

$H_{11}=\frac{a_{131}-a_{132}{\lambda_{1}}^{2}-a_{133}{\lambda_{1}}^{4}-a_{134}{\lambda_{1}}^{6}+a_{135}{\lambda_{1}}^{8}}{a_{136}-a_{137}{\lambda_{1}}^{2}+a_{138}{\lambda_{1}}^{4}-a_{139}{\lambda_{1}}^{6}-a_{140}{\lambda_{1}}^{8}}$,$H_{12}=\frac{a_{131}-a_{132}{\lambda_{2}}^{2}-a_{133}{\lambda_{2}}^{4}-a_{134}{\lambda_{2}}^{6}+a_{135}{\lambda_{2}}^{8}}{a_{136}-a_{137}{\lambda_{2}}^{2}+a_{138}{\lambda_{2}}^{4}-a_{139}{\lambda_{2}}^{6}-a_{140}{\lambda_{2}}^{8}}$,

$H_{13}=\frac{a_{131}-a_{132}{\lambda_{3}}^{2}-a_{133}{\lambda_{3}}^{4}-a_{134}{\lambda_{3}}^{6}+a_{135}{\lambda_{3}}^{8}}{a_{136}-a_{137}{\lambda_{3}}^{2}+a_{138}{\lambda_{3}}^{4}-a_{139}{\lambda_{3}}^{6}-a_{140}{\lambda_{3}}^{8}}$, $H_{14}=\frac{a_{131}-a_{132}{\lambda_{4}}^{2}-a_{133}{\lambda_{4}}^{4}-a_{134}{\lambda_{4}}^{6}+a_{135}{\lambda_{4}}^{8}}{a_{136}-a_{137}{\lambda_{4}}^{2}+a_{138}{\lambda_{4}}^{4}-a_{139}{\lambda_{4}}^{6}-a_{140}{\lambda_{4}}^{8}}$,

$H_{15}=\frac{a_{131}-a_{132}{\lambda_{5}}^{2}-a_{133}{\lambda_{5}}^{4}-a_{134}{\lambda_{5}}^{6}+a_{135}{\lambda_{5}}^{8}}{a_{136}-a_{137}{\lambda_{5}}^{2}+a_{138}{\lambda_{5}}^{4}-a_{139}{\lambda_{5}}^{6}-a_{140}{\lambda_{5}}^{8}}$,

$H_{21}=\frac{[a_{167}-a_{168}{\lambda_{1}}^{2}-{(a}_{169}+a_{170}{\lambda_{1}}^{2})(a_{150}+(a_{43}{\lambda_{1}}^{2}-a_{151}))]-{[(a}_{171}-a_{155}{\lambda_{1}}^{2})(a_{157}+(a_{43}{\lambda_{1}}^{2}-a_{158}))]}{(a_{166}+a_{162}{\lambda_{1}}^{2})((a_{164}+(a_{43}{\lambda_{1}}^{2}-a_{165}))}$,

$H_{22}=\frac{[a_{167}-a_{168}{\lambda_{2}}^{2}-{(a}_{169}+a_{170}{\lambda_{2}}^{2})(a_{150}+(a_{43}{\lambda_{2}}^{2}-a_{151}))]-{[(a}_{171}-a_{155}{\lambda_{2}}^{2})(a_{157}+(a_{43}{\lambda_{2}}^{2}-a_{158}))]}{(a_{166}+a_{162}{\lambda_{2}}^{2})(a_{164}+(a_{43}{\lambda_{2}}^{2}-a_{165}))}$,

$H_{23}=\frac{[a_{167}-a_{168}{\lambda_{3}}^{2}-{(a}_{169}+a_{170}{\lambda_{3}}^{2})(a_{150}+(a_{43}{\lambda_{3}}^{2}-a_{151}))]-{[(a}_{171}-a_{155}{\lambda_{3}}^{2})(a_{157}+(a_{43}{\lambda_{3}}^{2}-a_{158}))]}{(a_{166}+a_{162}{\lambda_{3}}^{2})(a_{164}+(a_{43}{\lambda_{3}}^{2}-a_{165}))}$,

$H_{24}=\frac{[a_{167}-a_{168}{\lambda_{4}}^{2}-{(a}_{169}+a_{170}{\lambda_{4}}^{2})(a_{150}+(a_{43}{\lambda_{4}}^{2}-a_{151}))]-{[(a}_{171}-a_{155}{\lambda_{4}}^{2})(a_{157}+(a_{43}{\lambda_{4}}^{2}-a_{158}))]}{(a_{166}+a_{162}{\lambda_{4}}^{2})(a_{164}+(a_{43}{\lambda_{4}}^{2}-a_{165}))}$,

$H_{25}=\frac{[a_{167}-a_{168}{\lambda_{5}}^{2}-{(a}_{169}+a_{170}{\lambda_{5}}^{2})(a_{150}+(a_{43}{\lambda_{5}}^{2}-a_{151}))]-{[(a}_{171}-a_{155}{\lambda_{5}}^{2})(a_{157}+(a_{43}{\lambda_{5}}^{2}-a_{158}))]}{(a_{166}+a_{162}{\lambda_{5}}^{2})(a_{164}+(a_{43}{\lambda_{5}}^{2}-a_{165}))}$,

$H_{31}=\frac{\left[ a_{173}+a_{176}H_{11}+a_{178}H_{21} \right]{\lambda_{1}}^{2}+[a_{175}H_{11}+a_{177}H_{21}-a_{172}]}{a_{174}}$,

$H_{32}=\frac{\left[ a_{173}+a_{176}H_{12}+a_{178}H_{22} \right]{\lambda_{2}}^{2}+[a_{175}H_{12}+a_{177}H_{22}-a_{172}]}{a_{174}}$,

$H_{33}=\frac{\left[ a_{173}+a_{176}H_{13}+a_{178}H_{23} \right]{\lambda_{3}}^{2}+[a_{175}H_{13}+a_{177}H_{23}-a_{172}]}{a_{174}}$,

$H_{34}=\frac{\left[ a_{173}+a_{176}H_{14}+a_{178}H_{24} \right]{\lambda_{4}}^{2}+[a_{175}H_{14}+a_{177}H_{24}-a_{172}]}{a_{174}}$,

$H_{35}=\frac{\left[ a_{173}+a_{176}H_{15}+a_{178}H_{25} \right]{\lambda_{5}}^{2}+[a_{175}H_{15}+a_{177}H_{25}-a_{172}]}{a_{174}}$,

$H_{41}=\frac{\left[ a_{41}{\lambda_{1}}^{2}-a_{39}-a_{42}H_{31}+a_{5}H_{11}+a_{6}H_{21} \right]}{a_{43}}$,$H_{42}=\frac{\left[ a_{41}{\lambda_{2}}^{2}-a_{39}-a_{42}H_{32}+a_{5}H_{12}+a_{6}H_{22} \right]}{a_{43}}$,

$H_{43}=\frac{\left[ a_{41}{\lambda_{3}}^{2}-a_{39}-a_{42}H_{33}+a_{5}H_{13}+a_{6}H_{23} \right]}{a_{43}}$,$H_{44}=\frac{\left[ a_{41}{\lambda_{4}}^{2}-a_{39}-a_{42}H_{34}+a_{5}H_{14}+a_{6}H_{24} \right]}{a_{43}}$,

$H_{45}=\frac{\left[ a_{41}{\lambda_{5}}^{2}-a_{39}-a_{42}H_{35}+a_{5}H_{15}+a_{6}H_{25} \right]}{a_{43}}$.

**Appendix III**

$$s_{1}=a_{23}{\lambda_{1}}^{2}+a_{24}\left( {\lambda_{1}}^{2}-e^{2} \right)-\left( 1+\tau_{1}\omega_{1}\omega\right)H_{31}-a_{25}\left( 1+\tau^{1}\omega_{1}\omega\right)H_{41}$$

$+a_{26}H_{11}+a_{27}H_{21}$,

$$s_{2}=a_{23}{\lambda_{2}}^{2}+a_{24}\left( {\lambda_{2}}^{2}-e^{2} \right)-\left( 1+\tau_{1}\omega_{1}\omega\right)H_{32}-a_{25}\left( 1+\tau^{1}\omega_{1}\omega\right)H_{42}$$

$+a_{26}H_{12}+a_{27}H_{22}$,

$$s_{3}=a_{23}{\lambda_{3}}^{2}+a_{24}\left( {\lambda_{3}}^{2}-e^{2} \right)-\left( 1+\tau_{1}\omega_{1}\omega\right)H_{33}-a_{25}\left( 1+\tau^{1}\omega_{1}\omega\right)H_{43}$$

$+a_{26}H_{13}+a_{27}H_{23}$,

$$s_{4}=a_{23}{\lambda_{4}}^{2}+a_{24}\left( {\lambda_{4}}^{2}-e^{2} \right)-\left( 1+\tau_{1}\omega_{1}\omega\right)H_{34}-a_{25}\left( 1+\tau^{1}\omega_{1}\omega\right)H_{44}$$

$+a_{26}H_{14}+a_{27}H_{24}$,

$$s_{5}=a_{23}{\lambda_{5}}^{2}+a_{24}\left( {\lambda_{5}}^{2}-e^{2} \right)-\left( 1+\tau_{1}\omega_{1}\omega\right)H_{35}-a_{25}\left( 1+\tau^{1}\omega_{1}\omega\right)H_{45}$$

$+a_{26}H_{15}+a_{27}H_{25}$,

$s_{6}=iea_{23}\sqrt{\frac{a_{44}}{a_{45}}}$, $s_{7}=2iea_{28}\lambda_{1}$,$s_{8}=2iea_{28}\lambda_{2}$,$s_{9}=2iea_{28}\lambda_{3}$,$s_{10}=2iea_{28}\lambda_{4}$,

$s_{11}=2iea_{28}\lambda_{5}$, $s_{12}=a_{28}\left( \frac{a_{44}}{a_{45}}+e^{2} \right)$, $s_{13}=a_{29}H_{11}\lambda_{1}+a_{30}H_{21}\lambda_{1}$,

$s_{14}=a_{29}H_{12}\lambda_{2}+a_{30}H_{22}\lambda_{2}$, $s_{15}=a_{29}H_{13}\lambda_{3}+a_{30}H_{23}\lambda_{3}$, $s_{16}=a_{29}H_{14}\lambda_{4}+a_{30}H_{24}\lambda_{4}$, $s_{17}=a_{29}H_{15}\lambda_{5}+a_{30}H_{25}\lambda_{5}$, $s_{18}=a_{31}H_{11}\lambda_{1}+a_{32}H_{21}\lambda_{1}$, $s_{19}=a_{31}H_{12}\lambda_{2}+a_{32}H_{32}\lambda_{2}$,

$s_{20}=a_{31}{H_{13}\lambda}_{3}+a_{32}H_{23}\lambda_{3}$, $s_{21}=a_{31}H_{14}\lambda_{4}+a_{32}H_{24}\lambda_{4}$, $s_{22}=a_{31}{H_{15}\lambda}_{5}+a_{32}H_{25}\lambda_{5}$,

$P=p^{*}e^{\left( \omega t+iey \right)}$.
